# Transdisziplinäres Expert:innen-Statement: Pflegeleitfaden für Menschen mit schwerem ME/CFS in der häuslichen Versorgung

**DOI:** 10.1007/s10354-026-01155-6

**Published:** 2026-06-01

**Authors:** Joachim Hermisson, Claudia Schreiner, Stefanie Weichselbaumer, Marlene Werner, Verena Hackl, Jacob Roth, Sandra Leiss, Anna Christina Maukner, Silvia Wojczewski, Astrid Hainzl, Sabine Hermisson, Kevin Thonhofer, Sabine Pleschberger, Kathryn Hoffmann

**Affiliations:** 1 Österreichische Gesellschaft für ME/CFS, Wien, Österreich; 2 Stiftungsprofessur Pflegewissenschaft, Abteilung für Primary Care Medicine, Zentrum für Public Health, Medizinische Universität Wien, Wien, Österreich; 3 Abteilung für Primary Care Medicine, Zentrum für Public Health, Medizinische Universität Wien, Wien, Österreich; 4 Fakultät für Mathematik und Max Perutz Labs, Universität Wien, Wien, Österreich; 5 MillionsMissing Deutschland, Bedburg-Hau, Deutschland

**Keywords:** ME/CFS, Schwere Myalgische Enzephalomyelitis/Chronisches Fatigue-Syndrom, Bettlägerigkeit, Pflege, Häusliche Versorgung, ME/CFS, Severe Myalgic encephalomyelitis/chronic fatigue syndrome, Bedridden, Nursing, Home-based care

## Abstract

**Hintergrund:**

Viele Betroffene von Myalgischer Enzephalomyelitis/Chronischem Fatigue-Syndrom (ME/CFS) sind hochgradig pflegebedürftig. Gleichzeitig bedingt die Post-Exertionelle Malaise (PEM), das Kernmerkmal von ME/CFS, dass bereits geringe körperliche, orthostatische, kognitive oder sensorische Belastungen eine disproportionale Zustandsverschlechterung auslösen können. Dadurch ergeben sich spezifische Anforderungen und erhebliche Herausforderungen in der häuslichen Versorgung. Die pflegerische Versorgung wird bislang überwiegend von Angehörigen getragen, die dabei nur unzureichend Hilfe und Unterstützung finden. Gleichzeitig bestehen erhebliche Defizite in Wissen, Versorgungsstrukturen und professioneller Orientierung für die an der Betreuung beteiligten Pflege- und Gesundheitsberufe sowie Ärzt:innen.

**Ziel:**

Ziel dieses Leitfadens ist es, pflegerische Maßnahmen so auszurichten, dass Überlastungen vermieden und Stabilität unterstützt wird.

**Methoden:**

Der Leitfaden basiert auf einer Zusammenstellung praxisorientierter Maßnahmen, die sich aus Sicht von Betroffenen und pflegenden Angehörigen bewährt haben. Diese wurden durch Expert:innen aus Pflegewissenschaft, Physiotherapie, Allgemeinmedizin und Public Health fachlich eingeordnet und inhaltlich weiterentwickelt.

**Ergebnisse:**

Der Leitfaden beschreibt Anpassungen zentraler Pflegedimensionen – von Ernährung und Körperpflege bis hin zu Kommunikation und Umgang mit emotionalen Belastungen – an krankheitsspezifische Belastungsgrenzen. Ergänzend werden Anforderungen an die Pflegebeziehung und die Planung von Hausbesuchen dargestellt sowie Möglichkeiten palliativer Pflegeprinzipien diskutiert.

## Abstract (en)

**Background:**

Many of those affected by myalgic encephalomyelitis/chronic fatigue syndrome (ME/CFS) have significant care needs. However, post-exertional malaise, the defining feature of ME/CFS, means that even minor physical, orthostatic, cognitive, or sensory stressors can trigger a disproportionate worsening of health status, condition and symptoms. This results in specific requirements and significant challenges in home care. Nursing care is still provided predominantly by family caregivers, who frequently lack adequate assistance and support. At the same time, there are significant gaps in knowledge, care infrastructure, and professional guidance for the nursing and healthcare professionals, as well as physicians, involved in providing care.

**Objective:**

The objective of this guide is to structure care measures in a way that prevents overexertion and promotes stability.

**Methods:**

The guide is based on a compilation of practice-oriented measures that have proven effective from the perspective of those affected and family caregivers. These were professionally categorized and further developed by experts in nursing science, physical therapy, general practice and public health.

**Results:**

The guide describes how to adjust key dimensions of care — from nutrition and personal hygiene to communication and managing emotional stress — to disease-specific exertion thresholds. Additionally, requirements for the caregiving relationship and the planning of home visits are outlined and the possibilities of palliative care principles are discussed.

## 1. Einleitung

Ziel pflegerischen Handelns ist die Ermöglichung eines würdevollen und bedarfsgerechten Lebens. Üblicherweise sind damit Aspekte wie Kommunikation, soziale Teilhabe, Aktivierung, Bewegung und Berührung verbunden. Bei Patient:innen mit Myalgischer Enzephalomyelitis/Chronischem Fatigue-Syndrom (ME/CFS) sind diese Prinzipien jedoch nur innerhalb der individuell sehr unterschiedlichen und stark fluktuierenden Belastbarkeit anwendbar, die durch die Erkrankung vorgegeben ist. Insbesondere bei schwer erkrankten Patient:innen ist die Belastungstoleranz extrem niedrig. Pflegemaßnahmen, die in anderen Versorgungskontexten als förderlich gelten, können hier zu einer deutlichen Zustandsverschlechterung führen und sind daher nicht anwendbar. Zentrales klinisches Merkmal ist die Post-Exertionelle Malaise (PEM), die bei ME/CFS eine zentrale Bedeutung für alle pflegerischen Entscheidungen hat [1–4].

Hieraus ergibt sich ein pflegerischer und medizinischer Ansatz, der sich grundlegend von etablierten, aktivierenden Pflegekonzepten unterscheidet. Er erfordert krankheitsspezifisches Wissen sowie eine ausgeprägte Sensibilität für individuelle Belastungsgrenzen. Dieser Zugang ist bislang jedoch kaum Bestandteil formeller Pflege- und medizinischer Ausbildungen und muss daher gezielt erlernt werden. Gleichzeitig ist die Belastung für Pflegende erheblich. Sie resultiert aus der extremen Versorgungssituation schwer erkrankter Patient:innen, die häufig eine 24-Stunden-Bereitschaft erfordert, sowie aus der Schwere und Dauer der Erkrankung und der ungewissen Prognose. Hinzu kommen oft mangelndes Krankheitsverständnis im sozialen Umfeld und eine unzureichende Unterstützung durch Gesundheits- und Sozialsysteme. Unter diesen Bedingungen geraten nicht nur die Betroffenen selbst, sondern auch pflegende Angehörige an die Grenzen ihrer Belastbarkeit und darüber hinaus.

In dieser Situation sind klar strukturierte Routinen und ein individuell angepasster Pflegeplan zentrale Instrumente, um sowohl Betroffenen als auch Pflegepersonen Sicherheit zu geben und ein medizinisch fundiertes Verständnis zu fördern. Trotz großer individueller Unterschiede ähneln sich viele pflegerische Herausforderungen, etwa in den Bereichen Ernährung, Mund- oder Körperpflege. Für diese Pflegedimensionen haben sich auf Grundlage praktischer Erfahrungen spezifische Maßnahmen und anpassbare Vorgehensweisen entwickelt.

Diese praxisbasierten Ansätze sind überwiegend außerhalb etablierter pflegerischer Versorgungskonzepte entstanden. Vor diesem Hintergrund kommt dem Erfahrungswissen von Betroffenen und pflegenden Angehörigen eine besondere Bedeutung zu. Es bildet die Grundlage für eine Orientierung an bewährten Vorgehensweisen (Best Practice) in einer bislang unzureichend abgedeckten Pflegesituation.

Der vorliegende Pflegeleitfaden greift dieses Erfahrungswissen auf. Er bündelt über Jahre hinweg gewonnene Erfahrungen und macht die daraus abgeleiteten Pflegedimensionen sowohl für die informelle als auch für die formelle Pflege nutzbar.

*Der Leitfaden erhebt keinen Anspruch auf Vollständigkeit, Rechtsverbindlichkeit oder universelle Anwendbarkeit. Er ist ausdrücklich nicht als rechtsverbindliche Handlungsvorgabe zu verstehen, sondern als fachlich-inhaltliche Orientierungshilfe für die pflegerische Praxis. Weder die Autor:innen noch die beteiligten Institutionen haften für unmittelbare oder mittelbare Schäden, einschließlich gesundheitlicher Beeinträchtigungen oder wirtschaftlicher Verluste, die durch die Umsetzung oder Unterlassung von Maßnahmen auf Basis dieser Empfehlung entstehen.*

## 2. Hintergrund und Entstehung des Leitfadens

Der Leitfaden wurde von der Österreichischen Gesellschaft für ME/CFS gemeinsam mit der Abteilung für Primary Care Medicine am Zentrum für Public Health der Medizinischen Universität Wien sowie der dort angesiedelten Stiftungsprofessur für Pflegewissenschaft erarbeitet. Ziel war es, evidenzbasiertes und erfahrungsbasiertes Wissen aus der professionellen Pflege zusammenzuführen und für pflegende Bezugspersonen von Menschen mit schwerer ME/CFS zugänglich zu machen.

International besteht weiterhin ein erheblicher Mangel an wissenschaftlicher Literatur zu ME/CFS, insbesondere auch im Bereich der pflegerischen Versorgung von schwer erkrankten Menschen. Betroffenenorganisationen berichten über ausgeprägte Versorgungslücken sowie über fehlendes Wissen im Gesundheitswesen. Die Patient:innen sind in ihrer Alltagsgestaltung stark eingeschränkt und weisen eine hohe Symptomlast auf. Ein erheblicher Teil ist pflegebedürftig und aufgrund mangelnder Anerkennung und fehlender struktureller Verankerung im Versorgungssystem fast ausschließlich auf die informelle Pflege durch An- und Zugehörige angewiesen. Diese wiederum sind weitgehend auf sich allein gestellt, Anleitungen und Hilfen sind kaum verfügbar.

### 2.1 Projektgruppe

Die Entwicklung des Leitfadens erfolgte in einer transdisziplinär zusammengesetzten Projektgruppe. Diese setzte sich aus Vertreter:innen der Österreichischen Gesellschaft für ME/CFS, Betroffenen, pflegenden Angehörigen sowie Fachpersonen aus Pflege, Pflegewissenschaft, Physiotherapie, Primary Care Medicine, Sozialmedizin und Public Health zusammen.

### 2.2 Population, Anwender:innen und Zielsetzung

#### Population

Die Empfehlungen des Leitfadens beziehen sich auf erwachsene Patient:innen (≥ 18 Jahre) mit schwerem ME/CFS.

Der Leitfaden ist grundsätzlich in unterschiedlichen Versorgungskontexten anwendbar. Ein besonderer Fokus liegt jedoch auf der häuslichen Versorgung, da die meisten schwer betroffenen Menschen mit ME/CFS ausschließlich zu Hause versorgt werden. Der Leitfaden konzentriert sich auf pflegerische Interventionen im Bereich des Symptommanagements, der Grundpflege sowie auf Maßnahmen im Umgang mit den krankheitsbedingten Belastungsgrenzen (Pacing).

#### Anwender:innen

Der Leitfaden richtet sich grundsätzlich an alle Personen, die formell oder informell in die Pflege und Betreuung von Menschen mit ME/CFS eingebunden sind oder diese veranlassen. In vielen Fällen erfolgt die alltägliche Versorgung überwiegend im häuslichen Umfeld durch Angehörige oder nahestehende Personen, weshalb diese eine zentrale Zielgruppe darstellen.

Gleichzeitig ist der Leitfaden anschlussfähig für professionelle Pflegeberufe und weitere Gesundheitsprofessionen sowie Hausärzt:innen, die im Rahmen von Hausbesuchen an der Versorgung beteiligt sind. Er kann dazu beitragen, ein besseres Verständnis für die spezifischen Bedürfnisse und Belastungen von Menschen mit ME/CFS und deren Angehörigen zu entwickeln, pflegerische Maßnahmen entsprechend anzupassen und Versorgungsbedingungen zu verbessern.

#### Ziel des Leitfadens

Ziel des Leitfadens ist es, zentrale Hinweise und Empfehlungen zur pflegerischen Versorgung von Menschen mit schwerem ME/CFS bereitzustellen. Er vermittelt grundlegende Informationen zum Krankheitsbild und formuliert praxisnahe Empfehlungen zu zentralen Pflegedimensionen des Alltags, darunter Ernährung, Körperpflege, Mobilität, Kommunikation und Symptommanagement.

Ein wesentliches Ziel besteht darin, vorhandenes wissenschaftliches Wissen, Erfahrungswissen von Betroffenen sowie praxisbezogene Perspektiven aus der Pflege zusammenzuführen und für die Versorgungspraxis zugänglich zu machen.

Die Empfehlungen des Leitfadens sind als Orientierungshilfe für pflegerische Entscheidungen zu verstehen und müssen stets im Kontext der individuellen Situation der Patient:innen angewendet werden.

### 2.3 Methodik

Dieser Leitfaden ist keine medizinische Leitlinie, sondern eine praxisorientierte Sammlung bewährter Maßnahmen aus Betroffenen- und Pflegefachperspektive. Er wurde in einem beteiligungsorientierten transdisziplinären Prozess entwickelt [5]. Ziel war die Erarbeitung einer Hilfestellung zur pflegerischen Versorgung von Menschen mit schwerem ME/CFS sowie die Generierung anschlussfähigen Wissens durch den Austausch der Projektpartner:innen. Er ersetzt nicht die individuelle pflegerische Verantwortung im jeweiligen Einzelfall. Die Anwendung der vorgeschlagenen Maßnahmen erfolgt in der alleinigen Verantwortung der pflegenden Person.

Die Rolle der wissenschaftlichen Partner:innen bestand insbesondere in der methodischen Beratung, der Strukturierung der Inhalte sowie der Einordnung vorhandener Literatur. Die inhaltliche Ausgestaltung der Empfehlungen wurde maßgeblich durch Betroffene und pflegende Angehörige geprägt.

Der Leitfaden unterscheidet sich von formal evidenzbasierten klinischen Leitlinien, orientiert sich jedoch in der Planung und Organisation stellenweise an etablierten Regelwerken (z. B. [6]). Entsprechend handelt es sich um eine strukturierte Zusammenstellung praxisrelevanter Empfehlungen aus Betroffenen- und Pflegefachperspektive, die soweit möglich durch vorhandene wissenschaftliche Literatur gestützt oder innerhalb der Projektgruppe konsensbasiert abgestimmt wurden.

Als Ausgangspunkt diente ein von der Österreichischen Gesellschaft für ME/CFS erstelltes Basisdokument, das im weiteren Verlauf gemeinsam mit Vertreter:innen der Pflegewissenschaft strukturell und inhaltlich weiterentwickelt wurde. Da sich viele Empfehlungen auf Aspekte der pflegerischen Grundversorgung beziehen, orientiert sich die Struktur des Leitfadens an etablierten pflegewissenschaftlichen Modellen und Klassifikationssystemen zur Beschreibung von Pflegedimensionen des Alltags.

Vorhandene wissenschaftliche Literatur zu ME/CFS sowie zur pflegerischen Versorgung wurde ergänzend gesichtet und in die Entwicklung der Empfehlungen einbezogen (wichtigste Ressourcen siehe Tab. 1).

Die Entwicklung des Leitfadens erfolgte in mehreren aufeinander aufbauenden Schritten, die charakteristische Merkmale transdisziplinärer Wissensgenerierung aufweisen [7]:

1. Konstituierendes Treffen der beteiligten Organisationen, Rollenklärung und Zeitplan
2. Definition von Zielgruppe, Anwender:innen sowie Zielsetzung des Leitfadens
3. Bildung einer Projektgruppe, mehrere Arbeitstreffen mit Diskussion und Aushandlungsprozessen zu Struktur, Inhalten und Literatur
4. Iterative Überarbeitung des Leitfadens und Abstimmung der Empfehlungen
5. Konsensbasierte Finalisierung der Inhalte innerhalb der Projektgruppe

#### Zeitrahmen und zukünftige Aktualisierung

Die Erarbeitung des Leitfadens erfolgte im Zeitraum zwischen November 2025 und März 2026. Eine Aktualisierung des Leitfadens ist vorgesehen, sobald neue relevante wissenschaftliche Erkenntnisse zur Versorgung von Menschen mit ME/CFS vorliegen.

## 3. Klinische Grundlagen

Dieses Kapitel stellt die für die Pflege zentralen klinischen Grundlagen von ME/CFS dar. Es umfasst krankheitsbestimmende Mechanismen wie die Post-Exertionelle Malaise (PEM) und das Prinzip des Pacings sowie häufige pflegerelevante Symptome, systemische Dysfunktionen und Komorbiditäten. Ziel ist es, die klinischen Zusammenhänge zu erläutern, die für Belastungssteuerung, Symptombeobachtung, Risikoeinschätzung und Priorisierung pflegerischer Maßnahmen maßgeblich sind.

### 3.1 Klinische Grundlagen von ME/CFS mit Relevanz für die Pflege

Myalgische Enzephalomyelitis/Chronisches Fatigue Syndrom (ME/CFS, ICD-10-GM G93.3) ist eine schwere, chronische Multisystemerkrankung. Ihr obligates Kernmerkmal ist die Post-Exertionelle Malaise (PEM). Die Prävalenz wird in verschiedenen Studien mit etwa 0,3–1 % der Bevölkerung angegeben, aktuelle Schätzungen auf Basis internationaler Studien und Daten zeigen eine Bevölkerungspävalenz von 0,7-0,8% für Österreich im Jahr 2024 [8]; im Zuge der COVID-19-Pandemie ist von einem deutlichen Anstieg auszugehen [1, 3]. Etwa ein Viertel der Betroffenen ist haus- oder bettgebunden [1]. Ein Teil dieser Patient:innen ist auf pflegerische Versorgung angewiesen.

ME/CFS tritt überwiegend postinfektiös auf und wird den postakuten Infektionssyndromen (PAIS) zugeordnet. Die Diagnose erfolgt anhand international konsentierter klinischer Kriterien (z. B. Kanadische Konsensuskriterien [CCC], [2, 9]). Zahlreiche biomedizinische Auffälligkeiten sind beschrieben; bislang steht jedoch kein einzelner, im klinischen Alltag routinemäßig einsetzbarer diagnostischer Biomarker zur Verfügung [2, 3].

Die Erkrankung betrifft das zentrale und autonome Nervensystem, das Immunsystem, das Gefäß- und Endothelsystem sowie die zelluläre Energieverfügbarkeit und -regulation [1, 10]. Charakteristisch ist eine pathologische Aktivitäts-Erholungs-Dysfunktion, die zu einer ausgeprägten und oft zeitverzögerten Zustandsverschlechterung nach Belastung führt (PEM, siehe Abschnitt 3.2).

Infolge dieser krankheitsbedingten Mechanismen verursacht ME/CFS erhebliche Einschränkungen der körperlichen, kognitiven und autonomen Funktionsfähigkeit. Das Spektrum reicht von deutlicher Alltagsbeeinträchtigung bis hin zu vollständiger Pflegebedürftigkeit. Schwere Verlaufsformen von ME/CFS gehören zu den gravierendsten funktionellen Beeinträchtigungen in der Medizin [4, 11, 12].

Die Literatur unterscheidet oft 4 Schweregrade von ME/CFS: mild, moderat, schwer und sehr schwer (z. B. [2]). Für die pflegerische Situation ist jedoch nicht die formale Schweregradkategorie entscheidend, sondern die individuelle Kombination aus Symptomprofil sowie Belastungs- und Reizintoleranz. Diese Faktoren schwanken oft stark im Krankheitsverlauf, zwischen besseren und schlechten Tagen und können auch von der Tageszeit abhängen. Aus diesem Grund und zugunsten des besseren Leseflusses wird in diesem Leitfaden nicht explizit zwischen schweren und sehr schweren Verläufen unterschieden. Im Fokus stehen alle pflegebedürftigen Patient:innen.

### 3.2 Post-Exertionelle Malaise (PEM)

Die Post-Exertionelle Malaise ist das zentrale und unverwechselbare Kennzeichen von ME/CFS [1, 9]. PEM beschreibt eine pathologische Aktivitäts-Erholungs-Dysfunktion, durch die es nach körperlicher, kognitiver, orthostatischer oder sensorischer Belastung zu einer unverhältnismäßigen und häufig zeitverzögert eintretenden Zustandsverschlechterung kommt. Eine ausgeprägte Manifestation dieser Zustandsverschlechterung wird als „Crash“ bezeichnet. Charakteristisch ist dabei nicht nur die Intensität der Reaktion, sondern vor allem die gestörte Regeneration: Der Körper ist nicht in der Lage, sich innerhalb eines physiologisch angemessenen Zeitraums von Belastungen zu erholen. Die Verschlechterung kann unmittelbar eintreten; typischerweise zeigt sich der wesentliche Effekt jedoch erst nach Stunden oder Tagen. Die Dauer eines solchen Crashs kann von mehreren Tagen bis zu Wochen reichen und in schweren Fällen auch zu einer irreversiblen Absenkung des Funktionsniveaus führen [2, 4, 9].

Die PEM ist Ausdruck der zugrunde liegenden multisystemischen Erkrankung und unterscheidet ME/CFS von primärer Dekonditionierung, unspezifischer Erschöpfung oder Belastungsreaktionen anderer Genese [1, 2]. Sie ist zentral für Diagnostik, Schweregradeinschätzung und alle pflegerischen Entscheidungen.

Ein durch PEM ausgelöster Crash kann bestehende Symptome deutlich verstärken und/oder neue Symptome hervorrufen. Die individuelle Toleranzschwelle ist dabei nicht statisch, sondern tagesabhängig und wird im Verlauf eines Tages wesentlich durch die vorangegangene Belastung bestimmt. Neben ausgeprägten Zustandsverschlechterungen (Crashs) berichten Betroffene klinisch auch über weniger anhaltende Verschlechterungen (Flare-ups). Diese erreichen in der Regel nicht das Ausmaß eines Crashs, können aber Vorboten sein und erfordern ebenso eine konsequente Belastungsreduktion (vgl. [2]).

Wird eine Überlastung frühzeitig erkannt und die Gesamtbelastung konsequent reduziert, kann sich der Zustand wieder auf das vorherige Funktionsniveau stabilisieren, eine vollständige Rückkehr ist jedoch nicht verlässlich vorhersehbar. Wiederholte Überlastung kann hingegen zu einer anhaltenden Zustandsverschlechterung und einer dauerhaften Absenkung der Toleranzschwelle führen, wodurch die Vulnerabilität für weitere Crashs steigt [1, 2, 4].

#### Konsequenzen für die Pflege

Ein zentrales Ziel pflegerischen Handelns bei ME/CFS ist daher die konsequente Vermeidung von Crashs. Dabei ist Folgendes zu beachten:

- **Überlastung ist nicht unmittelbar sichtbar. **ME/CFS-Patient:innen können auf Anforderung oder unter situativem Druck kurzfristig deutlich über ihre individuelle Belastungsgrenze hinausgehen. Da ein Crash häufig zeitverzögert eintritt, wird er nicht immer korrekt mit der auslösenden Belastung in Zusammenhang gebracht.
- **Im schweren Krankheitsspektrum können bereits minimale Reize überlasten.** Bei schwer Betroffenen liegt die Belastungstoleranz teilweise so niedrig, dass bereits basale Pflegehandlungen (z. B. Lagerungen, Nahrungsaufnahme, leise Kommunikation, schwaches Licht) einen Crash auslösen können [2, 4]. In solchen Fällen kann es zu wiederholten und sich überlappenden Crash-Phasen kommen (manchmal auch als „Rolling PEM“ bezeichnet; [4, 13]). Dabei kommt es zwischen einzelnen Belastungen nicht zu einer vollständigen Stabilisierung, sodass das Funktionsniveau fortlaufend niedrig und instabil bleibt.
- **Zustandsverschlechterungen sind multisystemisch. **Symptomverschlechterungen betreffen meist die individuellen Hauptsymptome (z. B. Schmerz, kognitive Einschränkungen, autonome Dysregulation), können jedoch auch neue Symptome hervorrufen (z. B. Tinnitus, der nur während eines Crashs auftritt). Sie sind nicht auf „Erschöpfung“ reduzierbar und korrelieren häufig nicht direkt mit der auslösenden Belastung (z. B. Muskelschmerzen nach kognitiver Überlastung).
- **Stabilisierung vor pflegerischer Optimierung. **Verschlechtert sich der Zustand kontinuierlich oder zeigt sich keine Stabilisierung, sollte nicht mit verstärkten Maßnahmen („mehr Unterstützung“) reagiert werden. Interventionen (z. B. zusätzliche Infusionen) können die Situation verschärfen. Stattdessen sollte die Gesamtbelastung konsequent reduziert werden. Dies kann bedeuten, Pflegehandlungen zu verkürzen, zu verschieben, auf ein absolutes Minimum zu begrenzen oder vorübergehend ganz auszusetzen.

### 3.3 Pacing als zentrales pflegerisches Prinzip

Pacing umfasst alle Maßnahmen und Strategien zur Steuerung von Aktivitäten, Anstrengungen und Reizen, mit dem Ziel, Überlastung und damit PEM-induzierte Crashs zu vermeiden.

Pacing ist keine Therapie im kurativen Sinne und führt nicht automatisch zu einer Verbesserung des Gesundheitszustands [2, 3]. Es ist jedoch die zentrale Bedingung, um den Zustand zu stabilisieren und damit günstige Voraussetzungen für eine mögliche Besserung im weiteren Verlauf zu schaffen.

Bei leicht bis moderat Erkrankten ist Pacing in erster Linie eine Form des Selbstmanagements im Rahmen des individuellen Toleranzbereichs. Dies setzt ein verständnisvolles Umfeld sowie geeignete soziale und strukturelle Rahmenbedingungen voraus, liegt aber in erster Linie in der Verantwortung der Betroffenen.

Bei Schwerkranken wird Pacing zunehmend zu einer Aufgabe für das Pflegeteam. Sämtliche pflegerische Maßnahmen (auch die in diesem Leitfaden beschriebenen) sind daraufhin zu prüfen, ob sie im konkreten Fall die Gesamtbelastung reduzieren oder unbeabsichtigt erhöhen. Dabei gilt:

- **Ziel ist nicht Aktivierung, sondern Stabilisierung**. Es ist nicht das Ziel, dass Betroffene möglichst viel selbst tun; vielmehr steht die Belastungsminimierung im Vordergrund: Sie sollten, sofern pflegerische Ressourcen vorhanden sind, möglichst viele Aufgaben abgeben können.
- **Belastungen summieren sich und müssen gegeneinander abgewogen werden.** Körperliche, kognitive, emotionale, orthostatische und sensorische Belastungen durch Aktivitäten oder Pflegehandlungen addieren sich und können sich gegenseitig verstärken. Jede Maßnahme verbraucht einen Teil der begrenzten Ressourcen. Pflegehandlungen sind daher stets im Kontext der Gesamtbelastung des Tages zu planen. Beispielsweise kann es an einem Tag mit einem Arztbesuch erforderlich sein, den gesamten restlichen Tag in strikter Ruhe zu verbringen, um einen Crash zu vermeiden.
- **Ruhephasen sind ein notwendiger Teil der Pflege.** Nach jeder pflegerischen Maßnahme sind ausreichend lange Ruhephasen erforderlich, möglichst reizarm, im Liegen und ohne zusätzliche Anforderungen. Besonders bei sehr schwer Erkrankten kann es notwendig sein, selbst kleine Pflegehandlungen über den Tag zu verteilen und zwischen einzelnen Schritten längere Pausen einzuplanen.
- **„Belastungsspielräume“ sind für Lebensqualität reserviert. **Potenzielle „Spielräume“ jenseits der notwendigen Pflegehandlungen sollten nicht ungeplant verbraucht werden. Diese begrenzten Reserven sind oft entscheidend für Lebensqualität und Selbstbestimmung, etwa für einen kurzen Kontakt, ein persönliches Ritual oder einen kleinen Moment der Normalität.

**Beispiel für fehlerhafte Pflege: **Eine Pflegeperson interpretiert eine kurzfristige Stabilisierung als Verbesserung und intensiviert beim Anreichen des Essens die Kommunikation. Die Patientin reagiert freundlich und antwortet, da es in diesem Moment noch möglich erscheint. Stunden später entwickeln sich starke Kopfschmerzen sowie eine ausgeprägte Licht- und Geräuschempfindlichkeit. In den folgenden Tagen ist sie so geschwächt, dass selbst wichtige stabilisierende Routinen, etwa der abendliche Besuch ihrer Katze, nicht mehr möglich sind. Dieses Beispiel verdeutlicht, dass selbst gut gemeinte Interaktionen eine relevante Zusatzbelastung darstellen können.

### 3.4 Pflegerelevante Symptome und Komorbiditäten

Charakteristisch für ME/CFS sind eine Reihe von Symptomen und Komorbiditäten, die die pflegerische Versorgung maßgeblich mitbestimmen. Sie können die Belastungstoleranz, die Reizverarbeitung, die Ernährungssituation, die Kreislaufstabilität sowie die Reaktion auf Medikamente und andere exogene Reize betreffen.

Die folgende Auswahl konzentriert sich auf Kernsymptome und für die pflegerische Praxis besonders relevante Komorbiditäten bei schweren Verläufen, wie in internationalen Leitlinien und klinischen Übersichtsarbeiten zu ME/CFS beschrieben [2–4]. Entscheidend sind stets die individuelle Ausprägung und Kombination.

#### 3.4.1 Reizintoleranz

Intoleranz gegenüber Licht, Geräuschen, Berührungen, Gerüchen und/oder chemischen Substanzen ist ein häufiges und bei schweren Verläufen oft extrem ausgeprägtes Merkmal von ME/CFS. Sie kann unterschiedliche sensorische Modalitäten gleichzeitig betreffen und im Verlauf erheblich schwanken. Bereits geringfügige Reize können als schmerzhaft, überwältigend oder vegetativ destabilisierend erlebt werden und eine PEM-induzierte Zustandsverschlechterung bis hin zu einem Crash auslösen. Diese Reizüberempfindlichkeit ist Ausdruck der gestörten neuroimmunologischen und vegetativen Regulation bei ME/CFS und darf nicht als „Überempfindlichkeit im psychologischen Sinn“ missverstanden werden.

Im schweren Spektrum sind Betroffene häufig nicht mehr in der Lage, sich eigenständig vor Reizen zu schützen. Selbst alltägliche Umgebungsreize wie leise Gespräche, diffuses Tageslicht, Küchengerüche, Vibrationen oder kurze Berührungen können eine relevante Belastung darstellen.

Viele schwer Betroffene zeigen außerdem eine ausgeprägte Sensitivität gegenüber Medikamenten. Bereits geringe Dosierungen können unerwartet starke oder paradoxe Reaktionen auslösen. Medikamente sind deshalb möglichst niedrig dosiert einzuschleichen („start low, go slow“). Neue Substanzen sollen einzeln und zeitlich versetzt eingeführt werden, um Reaktionen eindeutig zuordnen zu können.

Pflegehandlungen müssen unter möglichst reizarmen Bedingungen erfolgen. Reizreduktion ist keine Komfortmaßnahme, sondern eine zentrale Stabilisierungsvoraussetzung. Die Konsequenzen, die sich daraus für die pflegerische Praxis ergeben, sind vielfältig und beeinflussen sämtliche Pflegedimensionen, von Mobilität über Körperpflege bis hin zu Schlaf und medizinischer Versorgung. Praxisnahe Handlungsempfehlungen und hilfreiche Tipps werden in der Dimension „4.6 Sicherheit, Schutz und Infektionsprophylaxe“ aufgegriffen.

#### 3.4.2 Dysautonomie

Autonome Funktionsstörungen sind bei ME/CFS häufig und stellen insbesondere bei schweren Verläufen einen zentralen klinischen Problembereich dar. Die Dysregulation kann verschiedene Organsysteme betreffen und sich orthostatisch, gastroenterologisch, urogenital oder sudomotorisch manifestieren [1, 2, 12, 14, 15].

Klinisch dominieren orthostatische Formen der Dysautonomie. Besonders häufig finden sich Ausprägungen einer orthostatischen Intoleranz (OI), definiert als die Unfähigkeit, eine aufrechte Körperhaltung (Sitzen, Stehen, Kopfhochlagerung) ohne relevante Symptomverschlechterung zu tolerieren. Eine häufige Ausprägung ist das Posturale orthostatische Tachykardiesyndrom (PoTS), bei dem es beim Aufrichten zu einer pathologischen Herzfrequenzsteigerung kommt [2, 3, 14].

Bei ME/CFS mit komorbider OI kann eine aufrechte Körperposition nicht nur zu Schwindel, Übelkeit, Palpitationen, kognitiven Problemen oder Schwäche führen, sondern auch zu einem Crash durch orthostatische Belastung. In sehr schweren Fällen wird selbst eine leichte Kopfhochlagerung oder kürzeres Sitzen nicht toleriert. Dies kann mit erheblichen funktionellen Einschränkungen einhergehen und die pflegerische Versorgung maßgeblich beeinflussen:

- Lagerungswechsel langsam und schrittweise durchführen und nach orthostatischer Belastung eine ausreichende Erholungsphase einplanen.
- Nicht zwingend notwendige aufrichtende Positionierungen vermeiden. Kopfhochlagerung individuell prüfen und nicht routinemäßig anwenden.
- Pflegemaßnahmen zeitlich an Phasen besserer Kreislaufstabilität anpassen (z. B.: nachmittags statt morgens). Transfers sorgfältig vorbereiten und ggf. auf mehrere Etappen verteilen.
- Vitalzeichen bei orthostatischer Belastung beobachten (Herzfrequenz, Blutdruck), Flüssigkeits- und Elektrolytstatus berücksichtigen.
- Symptome nicht als „Angst“ oder „Dekonditionierung“ fehlinterpretieren.

##### Sturzrisiko und Sturzprophylaxe

Da bei OI plötzliche Schwäche, Schwindel und Kreislaufinstabilität auftreten können, besteht ein relevantes Sturzrisiko. Es ist jedoch zu differenzieren:

- Sehr schwer Erkrankte sind meist dauerhaft bettlägerig; hier liegt der Fokus weniger auf klassischer Sturzprophylaxe als auf sicherer Lagerung und Vermeidung lagerungsbedingter Zwischenfälle.
- Bei schwer Erkrankten, die noch Transfers durchführen oder zur Toilette gehen, besteht ein relevantes Sturzrisiko. Zu berücksichtigen ist aber, dass bei ME/CFS die Vermeidung von Crashs prioritäres Ziel ist. Präventive Maßnahmen dürfen nicht selbst zu zusätzlicher Belastung führen.
- Für eine kombinierte Sturz- *und* Crash-Prophylaxe Aufstehen oder Aufsetzen langsam, schrittweise und symptomgeleitet gestalten und bei angekündigtem Schwindel sofortige Rückkehr in die horizontale Position ermöglichen.

#### 3.4.3 Gastrointestinale Dysfunktionen und Mastzellaktivierung

Gastrointestinale Funktionsstörungen sind bei ME/CFS häufig und können die Versorgungssituation erheblich destabilisieren. Typische Symptome umfassen verzögerte Magenentleerung (Gastroparese), frühes Sättigungsgefühl, Übelkeit, Blähungen, Bauchschmerzen sowie ausgeprägte Nahrungsmittelunverträglichkeiten.

Zusätzlich finden sich bei einem Teil der Betroffenen Hinweise auf eine Mastzellüberaktivierung oder ein Mastzellaktivierungssyndrom (MCAS) [3, 16]. Hierbei kommt es zu einer inadäquaten oder überschießenden Freisetzung mastzellvermittelter Mediatoren (u. a. Histamin), mit multisystemischer, fluktuierender Symptomatik. Neben gastrointestinalen Beschwerden treten häufig Flush, Kreislaufreaktionen, Hautsymptome sowie eine erhöhte Sensitivität gegenüber Nahrungsmitteln, Medikamenten, Gerüchen oder chemischen Substanzen auf. Die Reaktionsbereitschaft kann stark variieren [17, 18]. Diese gastrointestinalen Funktionsveränderungen und Sensitivitäten können die Nahrungsaufnahme erheblich erschweren und zu Gewichtsverlust, Mangelernährung oder Dehydrierung führen.

Konsequenzen für die pflegerische Praxis:

- Individuelle Trigger (Medikamente, Gerüche, Temperatur, Stress) und Nahrungsmittelunverträglichkeiten identifizieren und systematisch erfassen.
- Nahrungsaufnahme zeitlich entzerren und ausreichend Ruhephasen einplanen. Grundsätzlich empfiehlt sich ein Schema mit 4–5 kleineren Mahlzeiten [19], eignet sich jedoch nicht für alle Betroffenen.
- Auf ausreichende Flüssigkeits- und Elektrolytzufuhr achten.
- Geruchs- und Reizbelastungen während der Mahlzeiten reduzieren.
- Bei bekannten schweren Reaktionen einen klar definierten Notfallplan bereithalten.

#### 3.4.4 Schmerzen

Schmerzen gehören zu den diagnostischen Kernkriterien von ME/CFS [9] und stellen bei schweren Verläufen eine erhebliche Belastung dar. Insbesondere können die körperlichen und vegetativen Belastungen starker Schmerzen Crashs auslösen, wodurch sich die Schmerzsymptomatik weiter verstärken kann. Eine solche Rückkopplung kann zu einer selbstverstärkenden Zustandsverschlechterung mit sich überlappenden Crash-Phasen („Rolling PEM“) führen und die vorrangige pflegerische Aufmerksamkeit erfordern.

Schmerzen können muskuloskelettal, neuropathisch, migräneartig oder belastungsinduziert auftreten und sind häufig fluktuierend. Bei einem Teil der ME/CFS-Betroffenen findet sich eine Small-Fiber-Neuropathie (SFN), also eine Schädigung der kleinen Nervenfasern, die unter anderem für Schmerz‑, Temperatur- und autonome Funktionen verantwortlich sind [20]. Klinisch können sich daraus neuropathische Schmerzen (brennend, stechend, elektrisierend), ausgeprägte Berührungsempfindlichkeit (Allodynie), Temperaturdysregulation, Schwitzen, Kreislaufinstabilität oder Missempfindungen ergeben [21].

Konsequenzen für die pflegerische Praxis:

- Schmerzsymptomatik ernst nehmen, nicht als Überempfindlichkeit fehlinterpretieren und insbesondere bei Gefahr einer selbstverstärkenden Zustandsverschlechterung konsequent behandeln.
- Bei Berührungsempfindlichkeit, Belastungen durch Druck und Reibung soweit möglich vermeiden. Berührungen durch einen kurzen, ggf. nonverbalen Hinweis vorbereiten, um Überraschungsreize zu verhindern.
- Behandlung unter ärztlicher Anleitung vor allem medikamentös (z. B. [3, 22]); *aber*: Unverträglichkeiten und Nebenwirkungen beachten.
- Komplementär eignen sich etwa schmerzlindernde Lagerungen (falls toleriert). Wenig geeignet ist z. B. eine Ablenkung durch andere Reize.

#### 3.4.5 Gelenkhypermobilität

Bei einem Teil der ME/CFS-Betroffenen wird eine generalisierte Gelenkhypermobilität im Sinn eines hypermobilen Ehlers-Danlos-Syndroms (hEDS) oder einer Hypermobilitäts-Spektrum-Störung (HSD) beschrieben [3]. Hier ist zu berücksichtigen, dass mechanische Instabilität bei hypermobilen ME/CFS-Patient:innen nicht nur (chronische) Schmerzen verursacht, sondern sekundär auch autonome Dysregulation begünstigen und somit PEM-assoziierte Zustandsverschlechterungen triggern oder verstärken kann [2, 3, 23–25]. Gelenkschutz und Stabilisierung sind daher als Bestandteil multimodaler und pflegerischer Maßnahmen zu berücksichtigen [24].

Hypermobilität betrifft nicht nur große Gelenke, sondern kann auch kleinere Strukturen (Kiefer, Rippen, Füße) einschließen. Bereits geringe Belastungen oder ungünstige Lagerungen können Schmerzen, Subluxationen oder Crashs auslösen. Bei einem Teil der Patient:innen mit ausgeprägter Bindegewebsschwäche wird eine Instabilität der Halswirbelsäule beschrieben, einschließlich einer kraniozervikalen Instabilität (CCI). Diese kann mit Nacken- und Kopfschmerzen, neurologischen Symptomen, Schwindel oder vegetativer Verschlechterung einhergehen [4].

Konsequenzen für die pflegerische Praxis:

- Achsengerechte, stabilisierende Lagerung aller Gelenke – insbesondere der Halswirbelsäule – unter Vermeidung forcierter Flexion, Extension oder Rotation.
- Keine Überstreckungen oder Zugbewegungen (z. B. am Kopf beim Umlagern).
- Bewegungen langsam, kontrolliert und in kleinen Bewegungsumfängen durchführen, passive Mobilisation nur mit großer Vorsicht.
- Bei Transfers auf zusätzliche Stabilisierung achten.
- Entlastende Hilfsmittel gezielt einsetzen (Kissen, Rollen, Schienen) und individuell verordnete Orthesen berücksichtigen.
- Hautfragilität beachten (Vorsicht bei Pflastern, Klebeelektroden).

Mechanische Instabilität und daraus resultierende schmerz- oder PEM-assoziierte Folgebelastungen sollten möglichst vermieden werden. Passive Mobilisation oder manuelle Techniken im Bereich hypermobiler Gelenke, insbesondere der Halswirbelsäule, sollten ausschließlich durch entsprechend qualifiziertes Fachpersonal (ggf. erfahrene Physiotherapeut:innen) und nur bei klarer Indikation erfolgen.

## 4. Pflegedimensionen bei ME/CFS

Die pflegerischen Herausforderungen bei schwer betroffenen Patient:innen sind vielfältig und betreffen mehrere Ebenen der Versorgung:

- Die ausgeprägte körperliche Schwäche kann so weit fortgeschritten sein, dass grundlegende Aktivitäten des täglichen Lebens nicht mehr selbstständig ausgeführt werden können. Dazu zählen unter anderem der Gang zur Toilette, das Halten eines Bechers, das Kauen sowie eigenständige Lageveränderungen im Bett [12].
- Die häufig extreme Reizsensibilität (siehe 3.4.1) kann dazu führen, dass pflegerische Maßnahmen nur unter stark reduzierten sensorischen Bedingungen möglich sind, bis hin zu Pflege in nahezu vollständiger Dunkelheit, bei minimalem Körperkontakt und maximaler Reizabschirmung [12, 18, 26–28].
- Schwere Formen der OI (siehe 3.4.2) schränken zahlreiche pflegerische Interventionen erheblich ein [12].
- Ausgeprägte Entkräftung sowie eine Intoleranz gegenüber kognitiven Reizen können die verbale Kommunikation einschränken oder vollständig unmöglich machen [12, 18, 26]. In einzelnen Fällen ist bereits die Anwesenheit einer weiteren Person im Raum nicht tolerierbar [27].
- Schwere Verläufe von gastrointestinalen Dysfunktionen und Nahrungsmittelunverträglichkeiten (siehe 3.4.3) können den zentralen pflegerischen Fokus darstellen, zeitweise vital bedrohlich werden und/oder eine enterale Sondenernährung erforderlich machen [12, 18].
- Hinzu kommen vielfältige Komplikationen durch Komorbiditäten sowie sekundäre gesundheitliche Problemlagen infolge von Bettlägerigkeit und der Schwere der Erkrankung, wie Immobilität, Infektanfälligkeit, Obstipation, Osteoporose oder Depressionen [12].

Die folgenden Abschnitte geben einen Überblick über typische Herausforderungen und mögliche pflegerische Maßnahmen in den einzelnen Pflegedimensionen, von Ernährung und Körperpflege bis hin zum Umgang mit emotionaler Belastung. Da bislang keine validen Studien zur pflegerischen Versorgung von Menschen mit ME/CFS vorliegen, basieren die dargestellten Empfehlungen auf dem praxisnahen Erfahrungswissen von Pflegepersonen und Betroffenen. Sie spiegeln damit den aktuellen Stand der Versorgungspraxis wider.

*Zu beachten ist, dass die Post-Exertionelle Malaise (PEM) und vorhandene Komorbiditäten alle Pflegedimensionen und Versorgungsbereiche maßgeblich mitbestimmen. Sämtliche Maßnahmen müssen individuell an die aktuelle Belastbarkeit sowie an bestehende Reizintoleranzen angepasst und somit am zentralen Prinzip des Pacing ausgerichtet werden (siehe 3.3).*

### 4.1 Essen und Trinken

Die Sicherstellung einer ausreichenden Energie- und Flüssigkeitszufuhr ist bei schweren ME/CFS-Verläufen oft eine pflegerische Herausforderung. Einerseits ist Untergewicht ein häufiges Problem. Andererseits kann die Nahrungsaufnahme selbst eine erhebliche körperliche Anstrengung darstellen und Crashs auslösen. Gastrointestinale Probleme oder Nahrungsmittelunverträglichkeiten können die Ernährung zusätzlich erschweren. Pflegeentscheidungen müssen in solchen Situationen sorgfältig zwischen notwendiger Versorgung und Belastungsreduktion austariert werden. Bei sehr schweren Verläufen oder in Krisensituationen kann das Abwägen zwischen gleichermaßen bedrohlichen Folgen notwendig sein. Eine strukturierte und verlässliche Sicherstellung der Nahrungs- und Flüssigkeitsaufnahme hat daher hohe Priorität. Idealerweise werden unterstützende Maßnahmen vorausschauend eingeleitet, bevor sie zwingend notwendig werden. Ist die Ernährung einigermaßen gesichert, stellt dies einen wichtigen Schritt zur Stabilisierung der Gesamtsituation dar.

#### Wesentliche Herausforderungen

Schwierigkeiten ergeben sich aufgrund mehrerer Faktoren, die oft gleichzeitig auftreten:

1. **Entkräftung und Muskelschwäche:** Essen ist häufig sehr anstrengend. Viele Patient:innen können aufgrund ausgeprägter Entkräftung ihre Arme nicht heben, nicht aufrecht sitzen oder den Kopf nicht halten. Selbst elementare Funktionen wie Kauen und Schlucken können eingeschränkt sein oder vollständig ausfallen [29].
2. **Orthostatische Intoleranz (OI, ggf. PoTS):** Im Extremfall tolerieren Betroffene selbst minimale Lageveränderungen oder ein leichtes Aufrichten im Bett nicht mehr [30] und müssen alle Nahrung und Getränke flach im Liegen zu sich nehmen.
3. **Nahrungsmittelunverträglichkeiten und -allergien** (oft im Rahmen einer Mastzellaktivierung oder eines MCAS): Diese erfordern häufig eine individuelle Anpassung der Ernährung [16].
4. **Gastrointestinale Beschwerden**: Motilitätsstörungen (mit Symptomen wie Schluckstörungen, Übelkeit, Verstopfung) bis hin zur Gastroparese können die Nahrungsaufnahme erheblich erschweren [29].
5. **Belastung durch Anwesenheit von Personen:** Wenn dies eine relevante Belastung darstellt, kann es das Anreichen von Nahrung oder Getränken deutlich erschweren oder unmöglich machen [27, 31].

#### Pflegerische Maßnahmen

Die geeigneten Maßnahmen hängen stark von Art und Schweregrad der Einschränkungen ab. Maßgeblich ist stets, welche Routine die geringste Belastung verursacht und gleichzeitig eine minimal ausreichende Versorgung sicherstellt. Manche Patient:innen können geeignete Mahlzeiten noch selbst zu sich nehmen. Bei schwerem Krankheitsverlauf oder in Crash-Phasen muss die Nahrung häufig angereicht oder über eine Sonde zugeführt werden.

- Feste Zeiten und Routinen schaffen Struktur, geben Sicherheit und reduzieren den kommunikativen Aufwand, müssen aber in Crash-Phasen flexibel angepasst werden.
- Idealerweise ist beim Essen und Trinken zumindest ein leichtes Aufsitzen oder Anheben des Oberkörpers möglich, da dies das Schlucken erleichtert und die Gefahr des Verschluckens vermindert. Wenn dies aufgrund von OI nicht toleriert wird, muss die Nahrungsaufnahme im Liegen erfolgen.
- Wenn Kauen möglich ist, können kleingeschnittene Speisen in Reichweite platziert werden. Feste, formstabile Speisen sind im Liegen besser kontrollierbar als flüssige oder breiige Konsistenzen. Unter geeigneten Bedingungen kann so auch eine selbstständige Nahrungsaufnahme möglich sein.
- Wenn das Kauen anstrengend wird, muss die Ernährung entsprechend angepasst werden. Für manche Betroffene sind kleingeschnittene, weiche Speisen (wie Brot ohne Rinde) noch eine Option, viele sind aber auf pürierte Kost oder Suppen angewiesen. Pürierte Speisen können auch die Verdauung erleichtern und bei Unverträglichkeiten individuell abgestimmt werden.
- Wenn Anstrengungen weiter reduziert und Mahlzeiten verkürzt werden müssen, sodass nur noch wenige Schlucke möglich sind, sollte auf eine hochkalorische Ernährung geachtet werden. Dabei kann kommerziell erhältliche Flüssignahrung hilfreich sein. Bei Unverträglichkeiten sind Zusammensetzung und Verträglichkeit sorgfältig zu prüfen.
- Bei starken Symptomen aufgrund von Allergien, MCAS oder Nahrungsmittelunverträglichkeiten empfiehlt sich ein schrittweiser Aufbau der Ernährung, ausgehend von einer individuell gut tolerierten, möglichst reizarmen Basisernährung. Eine fachliche Begleitung durch Diätolog:innen oder Allergolog:innen ist, wenn verfügbar, sinnvoll.
- Eine ausreichende Flüssigkeitszufuhr ist wichtig. Wenn möglich, sollte eine gut erreichbare Trinkflasche oder Schnabeltasse bereitstehen. Trinkflaschen sollten nur teilweise gefüllt sein, da volle Flaschen aufgrund ihres Gewichts eine zusätzliche Belastung darstellen. Lauwarmes Wasser wird häufig besser toleriert und spart Energie. Rehydrationslösungen können bei OI unterstützend wirken.

**Hilfsmittel und praktische Tipps**

- Wenn Betroffene selbst essen können oder wenn die Anwesenheit anderer belastender ist als die körperliche Anstrengung, helfen einfache Hilfsmittel wie leichtes Kunststoffgeschirr, Becher mit Trinkhalmen und rutschfeste Tabletts, die notwendige Energie für die Nahrungsaufnahme möglichst gering zu halten.
- Schnabeltassen unterstützen beim Anreichen oder selbstständigen Essen und Trinken und senken das Risiko einer Aspiration (Verschlucken). Bei ausgeprägten Schluckproblemen kann es hilfreich sein, Getränke zu verdicken. Dafür stehen spezielle Andickungsmittel zur Verfügung.
- Bei Histaminintoleranz kann die „Swiss Interest Group Histamine Intolerance(SIGHI)-Liste“ bei der Zusammenstellung einer geeigneten Diät hilfreich sein [32].
- Bei Allergien und Unverträglichkeiten auch auf Medikamentenfüllstoffe achten. Manche Apotheken bieten hypoallergene Rezepturen an.
- Rehydrationslösung kann auch selbst hergestellt werden (z. B. WHO-Lösung).

#### 4.1.1 Künstliche Ernährung

Bei sehr schweren Unverträglichkeiten oder ausgeprägten Problemen mit Kauen und Schlucken kann eine enterale Sondenernährung notwendig werden [27, 29, 31]. Sie sollte bei drohender Mangelernährung oder anhaltender unzureichender Nahrungsaufnahme frühzeitig ärztlich geprüft werden, nicht erst bei bereits eingetretenem kritischem Gewichtsverlust.

- In keinem Fall darf künstliche Ernährung vorenthalten oder hinausgezögert werden, um Betroffene auf diese Weise zum selbstständigen Essen zu bewegen [2, 29, 33].
- Eine nasogastrale Sonde kann im häuslichen Umfeld durch geschultes Fachpersonal gelegt werden und eine spürbare Entlastung ermöglichen, da sich Betroffene nicht mehr aktiv um die Nahrungsaufnahme sorgen müssen. Bei manchen Betroffenen wird eine Nasensonde jedoch aufgrund sensorischer Sensitivitäten im Gesichts- und Rachenbereich schlecht oder nicht toleriert. In solchen Fällen sollten alternative Formen der Ernährung frühzeitig mitbedacht werden.
- Andere Sonden (Perkutane endoskopische Gastrostomie [PEG], PEG‑J etc.) müssen im Krankenhaus gelegt werden und bringen zusätzlichen Pflegeaufwand mit sich, etwa hinsichtlich der Wundversorgung oder korrekter Medikamentengabe über die Sonde. Dennoch ist dies bei längerfristiger Sondenernährung oder bei sensorischer Unverträglichkeit einer nasogastralen Sonde unter Umständen der bessere Weg. Eine Schulung der betreuenden Pflegepersonen durch qualifiziertes Fachpersonal ist hierbei dringend empfohlen.
- Die enterale Sondennahrung muss individuell ausgewählt, behutsam eingeschlichen und die Verträglichkeit engmaschig beobachtet werden.
- Eine parenterale (intravenöse) Ernährung muss erwogen werden, wenn eine ausreichende enterale Ernährung aufgrund schwerer gastrointestinaler Funktionsstörungen nicht möglich ist. Indikationsstellung und Durchführung erfolgen ausschließlich ärztlich begleitet.

### 4.2 Ausscheidung und Toilettengang

Lösungen für den Toilettengang müssen an das individuelle Funktionsniveau und die aktuelle Toleranzgrenze angepasst werden. Für viele schwer betroffene Patient:innen ist der Toilettengang die größte, unvermeidbare Anstrengung des Tages. Entsprechend stellt er im Pflegeplan, neben Ernährung und Flüssigkeitsaufnahme, einen Schwerpunkt dar.

**Wesentliche Herausforderungen**

1. **Verdauungsprobleme** können durch autonome Dysregulation, Bettlägerigkeit oder Nebenwirkungen von Medikamenten bedingt sein und sowohl zu hartem als auch zu sehr weichem Stuhl führen.
2. **Längeres Sitzen** ist insbesondere bei ausgeprägter orthostatischer Intoleranz sehr belastend oder nicht tolerierbar.
3. Der eigenständige **Gang zur Toilette** – mehrfach täglich – ist für schwer Betroffene oft zu anstrengend oder gänzlich unmöglich. Licht- und Geräuschreize können zusätzlich belasten.
4. Die **Nutzung von Inkontinenz-Hilfsmitteln, wie Schutzhosen **im Bett, kann bei nicht inkontinenten Patient:innen auf starke psychische Barrieren stoßen.
5. Die pflegerische Unterstützung beim **Toilettengang** kann sehr aufwendig sein. Unregelmäßige Zeiten erfordern eine ständige Bereitschaft seitens der Pflegepersonen.
6. Der **Verlust der Selbstständigkeit** in diesem Bereich ist ein sensibles Thema und häufig mit Angst und Scham besetzt. Für viele Betroffene ist das emotional belastend.

#### Pflegerische Maßnahmen

Ziel ist – wie in allen Bereichen – größtmögliche Entlastung, wobei hier besonders auch psychische Aspekte zu berücksichtigen sind. Eine verlässliche Routine kann es Betroffenen ermöglichen, eine ansonsten zu belastende Prozedur dennoch zu tolerieren.

- Regelmäßige Zeiten sind von Vorteil und können, wenn möglich, eingeübt werden. Sie dürfen jedoch keine zusätzliche Energie kosten. Es empfiehlt sich eine sorgfältige Dokumentation, etwa zur Häufigkeit und Konsistenz. Diese Aufzeichnungen helfen dabei abzuschätzen, wann Maßnahmen ergriffen werden sollten bzw. diese zu evaluieren.
- Ein regelmäßiger, weicher Stuhlgang (ohne Diarrhoe) ist ein wichtiges Ziel, da er entlastet und Beschwerden vorbeugt. Wichtig ist eine ausreichende Flüssigkeitszufuhr. Außerdem kann, sofern individuell verträglich, eine ballaststoffreiche Ernährung hilfreich sein, etwa durch die Beimischung von Flohsamen oder Leinsamen (wenn ausreichend Flüssigkeit aufgenommen werden kann). Auch kleine Mengen Olivenöl (1–2 Esslöffel) sowie gut verträgliches Obst und Gemüse können unterstützend wirken. Blähende Speisen (z. B. Hülsenfrüchte, Kohl, Zwiebeln) sollten bei entsprechender Symptomatik reduziert werden [34].
- Reicht dies nicht aus, können Stuhlweichmacher (z. B. Macrogol) eingesetzt werden. Nur wenn auch damit kein ausreichender Effekt erzielt wird, sollten weitere Maßnahmen wie Einläufe oder stärkere Laxantien (Abführmittel) erwogen werden. Nach 2–3 Tagen ohne Stuhlgang sollten Gegenmaßnahmen ergriffen werden.
- Weniger geeignet sind komplementäre Anwendungen aus der Pflege, die aufgrund von sensorischen oder chemischen Reizen nur schwer toleriert werden, wie feuchtwarme Wickel/Bauchauflagen, ätherische Öle oder Bauchmassagen, sowie Lebensmittel, die gekaut werden müssen (z. B. getrocknete Feigen).
- Auch mobile Patient:innen sollten unterstützt werden, sobald der Toilettengang als anstrengend empfunden wird, z. B. durch Sitzgelegenheiten für Pausen auf dem Weg zur Toilette oder durch Hilfsmittel wie einen leichten Rollstuhl oder Rollator. Gedimmtes Licht reduziert die sensorische Reizbelastung. Ein Fußschemel kann die Darmentleerung erleichtern; feuchtes Toilettenpapier oder eine Intimdusche die Reinigung.
- Wird der Weg zur Toilette zu belastend, ist (falls möglich) ein Toilettenstuhl oder eine Campingtoilette im Raum oft die bessere Lösung, ggf. mit Hilfe beim Transfer. Eine Leibschüssel (und Urinflasche, v. a. bei Männern) ermöglicht die Toilette im Liegen. Da die Anwendung etwas Übung erfordert, sollte frühzeitig damit begonnen werden und nicht erst, wenn es nicht mehr anders geht.
- Die Nutzung von Inkontinenzhilfsmitteln (Pants, Schutzhosen) kann bei schwerem ME/CFS auch dann notwendig werden, wenn keine Inkontinenz vorliegt, sondern der Toilettengang aufgrund der Schwere der Erkrankung nicht mehr durchführbar ist. Für viele Betroffene stellt dies eine hohe psychische und praktische Barriere dar (d. h., es funktioniert nicht, selbst wenn sie wollen), insbesondere wenn solche Hilfsmittel erstmals in einer akuten Überlastungssituation eingesetzt werden müssen. Ein behutsames, vorausschauendes Vorgehen, klare Routinen sowie die Wahrung von Würde und Autonomie sind daher besonders wichtig.
- Ein Blasendauerkatheter kann in speziellen Fällen hilfreich sein, z. B. wenn die Anwesenheit einer Pflegeperson nur schwer toleriert wird oder aufgrund autonomer Dysfunktion eine Blasenfunktionsstörung vorliegt [15]. Da das Legen, Tragen und Reinigen bei Reizempfindlichkeit belastend sein kann und durch den Katheter ein erhöhtes Infektionsrisiko besteht, ist eine sorgfältige Abwägung notwendig. Eine Anleitung durch geschultes Pflegepersonal ist erforderlich [35].
- Der genaue Ablauf bei der Reinigung im Bett sollte einer festen „Choreografie“ folgen, an die sich alle Beteiligten strikt halten (wann Unterlage auflegen, auf die Seite drehen etc.).

**Hilfsmittel und praktische Tipps**

- Bei Benutzung einer Bettpfanne oder gelegentlichem Urinverlust ist ein Nässeschutz für die Matratze wichtig. Bewährt hat sich eine Kombination aus wasserdichtem Schutz direkt auf dem Matratzenkern und einem darüberliegenden waschbaren Matratzenbezug oder Topper, der Feuchtigkeit aufnimmt.
- Einmal-Slips mit Aufnahmefunktion können bei Menstruationsbeschwerden oder leichter Inkontinenz hilfreich sein. Manche Frauen nutzen spezielle Urinierhilfen, um im Sitzen oder Liegen eine Harnflasche benutzen zu können.
- Geruchsbelastungen können, sofern toleriert, durch spezielle geruchsneutralisierende Produkte reduziert werden. Duftstoffe, Raumdüfte oder parfümierte Produkte sind häufig nicht verträglich und sollten vermieden werden.
- Eine Campingtoilette kann die Eigenständigkeit erhalten, wenn der Weg zur Toilette zu anstrengend ist. Campingtoiletten müssen nur alle 1–2 Tage geleert werden. Chemische Zusätze können die Intervalle verlängern, sind jedoch nicht immer verträglich.

### 4.3 Körperpflege und Kleiden

In der professionellen Pflege ist „sichtbare bzw. vollständige Sauberkeit“ häufig implizit mit guter Versorgung verknüpft. Dieses Verständnis ist bei ME/CFS kritisch zu hinterfragen. „Perfekte“ oder „vollständige“ Körperpflege darf nicht zum Maßstab „guter Pflege“ werden, wenn sie im Widerspruch zu Belastungsgrenzen steht.

Ein gewisses Maß an Sauberkeit und Körperpflege ist medizinisch indiziert, trägt wesentlich zum subjektiven Wohlbefinden bei und spielt eine wichtige Rolle im Erhalt von Würde, Identität und Selbstwert von Betroffenen. Gleichzeitig steht bei schwerem ME/CFS der Schutz vor Zustandsverschlechterung im Vordergrund, selbst wenn dies eine Reduktion gewohnter Pflegehandlungen erfordert.

Zentral ist daher, gemeinsam mit den Betroffenen herauszufinden, welche Pflegehandlungen tatsächlich toleriert werden. Dabei ist zu berücksichtigen, dass Betroffene ihre Belastungsgrenzen aus Scham, Gewöhnung oder Rücksichtnahme mitunter überschätzen oder zu hoch angeben. Der Verzicht auf gewohnte Pflegeroutinen (z. B. regelmäßiges Haarewaschen) kann zudem emotional belastend sein. Pflegepersonen sollten den Anpassungsprozess aktiv begleiten und versuchen, ihn möglichst zu normalisieren. Sie sollten dabei auf Zeichen einer Überlastung achten (veränderte Atmung, Blässe, verlangsamte Reaktion) und reagieren. In der Praxis sind sie öfter „Anwält:innen des Pacings“ als „Garanten der Sauberkeit“. Ziel ist nicht das Aufrechterhalten äußerer Standards, sondern der Schutz vor klinischer Verschlechterung bei gleichzeitiger Wahrung von Würde.

**Wesentliche Herausforderungen**

1. **Duschen und Haarewaschen **stellen für viele Betroffene eine erhebliche körperliche Belastung dar (orthostatische Beanspruchung, Temperaturwechsel, sensorische Reize).
2. **Ausgeprägte Berührungsempfindlichkeit** kann Körper- und Haarpflege erheblich erschweren und zeitweise unmöglich machen.
3. **Regelmäßige Nagelpflege und Rasur **sind besondere Herausforderungen.
4. **Pflege durch andere Personen und eine reduzierte Körperpflege **kann als Verlust von Autonomie, Intimität und Würde erlebt werden und psychisch stark belasten.

#### Pflegerische Maßnahmen

Bei schwerem ME/CFS orientiert sich die Körperpflege nicht an Vollständigkeit, sondern an medizinischer Notwendigkeit und individueller Belastungstoleranz. Grundlage hierfür sind allgemeine pflegewissenschaftliche Standards zur Sicherung der Haut- und Schleimhautintegrität sowie zur Infektionsprophylaxe [36]. Gleichzeitig betonen ME/CFS-spezifische Leitlinien die konsequente Orientierung an den Belastungsgrenzen, um post-exertionelle Zustandsverschlechterungen zu vermeiden [1, 2]. Ein angepasstes Minimalprogramm in der Körperpflege verfolgt drei Ziele:

1. Infektionsprophylaxe (Intimhygiene, Mundhygiene, Reinigung bei Kontamination)
2. Erhalt der Hautintegrität (wie Dekubitus- und Entzündungsprophylaxe)
3. Wahrung von Würde bei maximaler Belastungsreduktion

Alle darüber hinausgehenden Maßnahmen sind individuell abzuwägen und nur dann durchzuführen, wenn sie toleriert werden.

- Arbeitsschritte gut vorbereiten, um die Aufenthaltsdauer im Zimmer zu verkürzen. Pflegehandlungen priorisieren sowie ruhig, strukturiert und zeitlich begrenzt durchführen.
- Teilkörperwäsche im Bett ist für schwer Betroffene die schonendste Variante. Hier kann auch „Minimalwäsche“ (Gesicht, Hände, Intimbereich) oder oberflächliches Waschen mit einem feuchten Tuch/Waschlappen ausreichen. Anstrengungen, wie Arme heben oder im Bett drehen, sollten nur im sicheren Toleranzbereich erfolgen.
- Moderat Betroffene können ggf. mit einer Sitzhilfe und Unterstützung noch duschen. Beim Transfer ins Bad ist insbesondere bei Entkräftung und OI auf Sturzgefahr zu achten (siehe 3.4.2).
- Eine regelmäßige Hautinspektion ist insbesondere an gefährdeten Hautarealen durchzuführen: Sakralbereich, Fersen, Ellenbogen, Schulterblätter, Hinterkopf und Hautfalten [36]. Sie sollte möglichst im Rahmen ohnehin notwendiger Pflegehandlungen erfolgen, um zusätzliche Belastung zu vermeiden.
- Dekubitus (Wundliegen) tritt bei ME/CFS trotz Bettlägerigkeit nach klinischer Erfahrung seltener auf als bei vielen anderen immobilisierenden Erkrankungen, muss aber dennoch im Blick behalten werden. Eine anhaltende, nicht wegdrückbare Rötung (verblasst nicht bei leichtem Druck) stellt bereits eine Gewebeschädigung dar und erfordert unmittelbare Druckentlastung [36]. Bei Verdacht auf beginnende Schädigung frühzeitig fachliche Einschätzung einholen.
- Anhaltende Feuchtigkeit (durch Schwitzen, Inkontinenz oder längeren Kontakt mit feuchten Materialien) kann zu einer Aufweichung der Haut (Mazeration) führen. Mazerierte Haut ist anfälliger für Infektionen, da sie einen günstigen Nährboden für Bakterien und Pilze bietet. Die Haut sollte nach der Reinigung sanft trockengetupft (nicht gerieben) werden. Insbesondere in den Hautfalten auf vollständige Trocknung achten [36].
- Hautschutz: Eincremen nur, wenn gewünscht oder medizinisch indiziert. Bei übermäßiger Feuchtigkeit (z. B. durch starkes Schwitzen) können zwischen den Hautfalten Barriereschutzprodukte (Zinkoxid) angewendet werden. Bei sehr trockener Haut pH-neutrale und unparfümierte Produkte verwenden [36]. Grundsatz: so wenig wie möglich, so viel wie nötig.
- Haarewaschen im Bett (z. B. mit einer Waschhaube oder aufblasbaren Wanne) nur, wenn ausdrücklich gewünscht und problemlos toleriert.
- Haare/Bart in größeren Abständen (Wochen oder Monate) kürzen. Dafür sollten möglichst stabile Phasen genutzt werden, ggf. auf mehrere kurze Einheiten verteilen. Haare möglichst kurz zu halten, erleichtert die Pflege erheblich.
- Finger- und Zehennägel regelmäßig kontrollieren (Infektionen/Pilze/Verletzungsgefahr) und zumindest in größeren Abständen kürzen (ggf. etappenweise nur einzelne Nägel pro Tag). Bei sehr harten oder eingewachsenen Nägeln ggf. professionelle Fußpflege hinzuziehen.

**Hilfsmittel und praktische Tipps**

- Hilfsmittel für das Haareschneiden (Rasierer, elektrischer Rasierer oder (Bart‑)Schere) sollten den individuellen Bedürfnissen angepasst werden, je nachdem, ob die Dauer, Geräusche oder Berührungsreize als besonders anstrengend empfunden werden.
- Kämmen und Kopfhautpflege: Kurze Haare benötigen meist kein Kämmen. Bei juckender Kopfhaut können weiche Massagebürsten hilfreich sein.
- Beim Haarewaschen kann eine Ablage oder Unterstützung für den Kopf (z. B. Badewannenauflage) die Belastung reduzieren.

#### 4.3.1 Mundpflege

Zahn- und Mundprobleme sind bei schwerem ME/CFS ein häufiges und pflegerisch relevantes Folgeproblem, insbesondere bei stark eingeschränkter Selbstversorgung und erschwertem Zugang zu zahnärztlicher Versorgung. Eine unzureichende Mundpflege kann zu oralen Infektionen, Karies und Schmerzen führen. Entzündliche Prozesse im Mundraum sind zudem mit systemischen Auswirkungen assoziiert [37, 38]. Mundpflege ist daher pflegerisch notwendig und hat bei schwerem ME/CFS einen besonders hohen Stellenwert. Gleichzeitig stellt auch die Zahnhygiene selbst eine relevante Belastung dar.

**Wesentliche Herausforderungen**

1. **Zähneputzen ist häufig erheblich belastend**, insbesondere bei gründlicher Durchführung. Licht, Geräusche und Vibrationen können zusätzlich belasten.
2. **Eine Übernahme der Zahnhygiene durch Pflegende erfordert Übung**. Es kann Zeit benötigen, bis eine gute Routine entwickelt ist.
3. **Weitergehende zahnärztliche Behandlungen** (z. B. Füllungen) sind für schwer Betroffene meist nicht verfügbar.

##### Pflegerische Maßnahmen

Auch in der Mundpflege orientieren sich Umfang und Intensität an medizinischer Notwendigkeit und individueller Belastungstoleranz.

- Für die Mundpflege im Bett kann das Kopfteil kurz angestellt werden; ausgespuckt wird in eine Nierenschale oder ein geeignetes Gefäß.
- Gründliches Zähneputzen einmal täglich (idealerweise abends) ist sinnvoller als mehrfaches oberflächliches Putzen. Fluoridhaltige Zahnpasta (wenn verträglich) wird aus zahnmedizinischer Sicht empfohlen [39]. Antibakterielle Mundspülungen können ergänzend eingesetzt werden, sofern sie gut vertragen werden. Interdentalbürsten oder Zahnseide nur, wenn sie toleriert werden.
- Ein festes Schema (z. B. innen/außen, oben/unten) kann helfen, Kommunikation während des Putzens zu minimieren. Sofern möglich, sollte der Mundraum regelmäßig auf Rötungen, Beläge, Druckstellen oder Infektionszeichen inspiziert werden.
- Je nach Bedarf kann auch eine Aufgabenteilung gestaltet und in die Routine aufgenommen werden. Manchmal ist es z. B. sinnvoll, dass Betroffene bestimmte Bereiche selbst nachreinigen, da sie ein besseres Gefühl für verbleibende Beläge haben, insbesondere bei schwachem Licht.
- Wenn möglich, zahnärztliche Hausbesuche für Sichtkontrollen organisieren. Bei Verdacht auf Karies oder Entzündung sollte frühzeitig gehandelt werden, da spätere Behandlungen deutlich belastender sein können. In frühen Stadien kann eine intensive Fluoridierung hilfreich sein; geeignete Präparate sollten mit dem zahnärztlichen Fachpersonal besprochen werden.

**Hilfsmittel und praktische Tipps**

- Wenn sie toleriert werden, können elektrische Zahnbürsten die Reinigung erleichtern. Ultraschallzahnbürsten sind leiser, vibrieren aber stärker; entscheidend ist die individuelle Verträglichkeit.
- Bei Mundtrockenheit helfen häufige kleine Schlucke Wasser, zuckerfreie Lutschpastillen und ggf. Befeuchtungsgels oder Speichelersatzprodukte.
- Pflegepersonen ohne Erfahrung in der assistierten Mundpflege sollten sich vorab mit Technik und Ablauf vertraut machen und wenn möglich an Freiwilligen üben.

#### 4.3.2 Schlafanzug und Bettwäsche

Der Wechsel von Schlafanzug und Bettwäsche kann für ME/CFS-Betroffene sehr belastend sein. Feste Routinen und ein nicht zu häufiger Wechsel können entlasten.

**Wesentliche Herausforderungen**

1. Das **An- und Ausziehen eines Schlafanzugs **erfordert Heben und Bewegen der Extremitäten sowie Wenden im Bett.
2. Der **Wechsel des Bettlakens mit Patient:in im Bett** erfordert eine eingeübte Routine und mehrfaches Wenden. Decken und Polster (Kissen) lassen sich leichter separat wechseln.
3. Besonders schwierig ist ein Wäschewechsel bei **Berührungsempfindlichkeit.**
4. **Gestörte Thermoregulation** (zu heiß/kalt) kann häufiges Anpassen der Decken erforderlich machen.

**Pflegerische Maßnahmen**

- Oberteile ggf. hinten öffnen (wie ein Flügelhemd) und im Nacken mit Bändern oder Verschlüssen versehen. So ist ein Wechsel ohne Umdrehen und (fast) ohne Heben der Arme möglich. Oberteile je nach Verträglichkeit täglich oder in größeren Abständen wechseln.
- Unterteile weit genug wählen und am besten beim Toilettengang wechseln. Einteiler und Nachthemden sind unpraktisch. Auf Unterwäsche kann meist verzichtet werden. Falls doch notwendig (z. B. während der Menstruation), können Slips mit Verschlüssen hilfreich sein.
- Bettlakenwechsel: Nur wenn notwendig und vom Zustand her toleriert. Die Routine sollte an die Gegebenheiten angepasst werden. Typische Vorgehensweise: vorsichtiges Drehen auf eine Seite, teilweises Entfernen und Vorbereiten des frischen Lakens, anschließend kontrolliertes Drehen über die mittig gefalteten Laken und Abschluss des Wechsels. Wenn kurze Zeiten außerhalb des Bettes möglich sind (z. B. Toilettenstuhl), sollten diese genutzt werden. Bei eingespielter Routine kann ein Lakenwechsel mit zwei Personen sehr zügig (innerhalb von etwa 30 s) erfolgen.
- Frisch bezogene Decken und Kopfkissen bereithalten, außerhalb des Zimmers vorbereiten.
- Bei Kleidung und Bettwäsche auf individuell gut verträgliche Materialien achten, besonders im Hinblick auf sensorische Empfindlichkeit und gestörte Thermoregulation. An den Füßen können bei Bedarf und sofern gut toleriert, weiche Bettsocken verwendet werden.

### 4.4 Ruhen und Schlafen

Schlafstörungen sind ein zentrales Merkmal von ME/CFS [1, 2] und zeichnen sich insbesondere durch nicht erholsamen Schlaf, aber auch starke Ein- und Durchschlafprobleme aus. Typisch ist außerdem eine oft erhebliche Verschiebung des Tag-Nacht-Rhythmus mit Schlafphasen bis in den späten Vormittag und wachen Phasen bis weit nach Mitternacht. Bei schwer Betroffenen verschärft sich die Situation dadurch, dass neben dem Schlaf häufig nur „waches Ruhen“ im abgedunkelten Zimmer möglich ist. Das Bett ist damit nicht nur Schlafort, sondern nahezu durchgehend Lebensraum. Trotz der eingeschränkten Erholungsfunktion bleibt ausreichender Schlaf ein wichtiges Ziel im Symptommanagement. Auch nicht erholsamer Schlaf ist gegenüber anhaltender Schlafdeprivation vorzuziehen und kann zur Stabilisierung des Zustands beitragen.

**Wesentliche Herausforderungen**

1. Ein stark **verschobener Tag-Nacht-Rhythmus** kann die Pflegeplanung erschweren, vor allem wenn regelmäßige Pflegehandlungen (Essensgaben, Toilette) am späten Abend bis/nach Mitternacht notwendig sind.
2. **Schlafstörungen** bei ME/CFS sind nicht primär verhaltensbedingt, sondern werden mit einem komplexen Zusammenspiel autonomer Dysregulation, neuroinflammatorischen Prozessen, Mastzellaktivierung und Schmerzsymptomatik in Verbindung gebracht [4]. Dementsprechend sind allgemeine schlafhygienische Maßnahmen in der Regel unzureichend.
3. Das **Bett** ist die Umgebung, in der schwer Betroffene nicht nur die Nacht, sondern häufig 95–100 % des Tages verbringen. Es muss den unterschiedlichen Bedürfnissen gerecht werden und insbesondere eine **schonende Pflege** für Patient:in und Pflegende ermöglichen.

#### Pflegerische Maßnahmen

Klassische Schlafhygieneempfehlungen (z. B. „nur zum Schlafen ins Bett“, „tagsüber nicht ruhen“) sind bei schwerem ME/CFS oft kontraindiziert oder nicht umsetzbar. Stattdessen stehen folgende Ziele im Vordergrund:

- Pflegeplanung soweit möglich an die individuellen Schlafphasen anpassen. Einen stabilen individuellen Rhythmus akzeptieren und nicht aktiv versuchen, den verschobenen Tag-Nacht-Rhythmus zu normalisieren.
- Eine vertraute Abendroutine (z. B. eine feste Reihenfolge der Abendpflege oder ein kurzes persönliches Ritual) vermittelt Abschluss und Sicherheit und kann den Übergang vom wachen Ruhen in den Schlaf erleichtern.
- Für eine individuell angenehme und möglichst konstante Raumtemperatur sorgen.
- Viele Betroffene benötigen schlafunterstützende Medikamente. Indikationsstellung sowie Auswahl erfolgen ärztlich. Pflegende unterstützen bei der Beobachtung von Wirkung und Verträglichkeit. Übersichten zu in der ME/CFS-Praxis verwendeten Substanzen finden sich in spezialisierten Behandlungsempfehlungen (z. B. [3, 22]).
- Bei vollständiger Bettlägerigkeit unterstützt ein höhenverstellbares Pflegebett eigenständige Positionsanpassungen und ermöglicht sowohl für Betroffene als auch für Pflegepersonen eine schonendere Pflege. Ein elektrisch verstellbarer Lattenrost mit Fernbedienung bietet eine provisorische Lösung.

**Hilfsmittel und praktische Tipps**

- Notwendige Utensilien (z. B. Wasserflasche oder Schnabeltasse, Klingel) sollten verlässlich so positioniert werden, dass sie leicht erreichbar sind. Ein breiteres Bett hilft, die Ablagefläche zu vergrößern.
- Bei einer als unbequem empfundenen Matratze eignet sich ein (ggf. auch waschbarer) Matratzentopper als erste flexible Maßnahme.
- Eine gut sichtbare Uhr und ggf. ein Kalender helfen bei der zeitlichen Orientierung.

### 4.5 Mobilität, Lagerung und Transport

Pflegebedürftige ME/CFS-Patient:innen sind fast immer hausgebunden und meist dauerhaft bettlägerig. Mobilität im klassischen Sinn ist hier nicht Ziel der Pflege, sondern stellt einen hohen Belastungs- und Risikofaktor dar. Je nach Situation und Schweregrad können Betroffene regelmäßige Transfers (Bad, Toilette, zwischen Zimmern) manchmal aber noch selbst durchführen. Zu beachten ist, dass bei dauerhafter Immobilität und Bettlägerigkeit ein erhöhtes Risiko für Kontrakturen, Spitzfußentwicklung und Dekubitus besteht. Auch bei Bettlägerigkeit können Situationen eintreten, etwa notwendige medizinische Eingriffe, bauliche Maßnahmen oder Gefährdungslagen (z. B. Schimmel, Brand), die einen Ortswechsel unvermeidbar machen. Die Belastung sollte in solchen Fällen ebenfalls möglichst gering gehalten werden, auch wenn ein Crash nicht immer vermieden werden kann.

**Wesentliche Herausforderungen**

1. Regelmäßige **Lagerungen** bei einem relevanten Dekubitusrisiko und zur Kontraktur- und Spitzfußprophylaxe stellen eine Herausforderung dar, da Positionswechsel selbst Belastungsreaktionen verursachen können.
2. Viele Betroffene sind aufgrund **extremer Entkräftung** und/oder physischer Intoleranz nicht eigenständig mobil, auch nicht mithilfe eines Rollstuhls.
3. Patient:innen mit **ausgeprägter OI** (z. B. bei PoTS) können nur liegend transportiert werden.
4. Bereits das **Verlassen der geschützten Umgebung** (Bett, Pflegezimmer) kann eine Zustandsverschlechterung bis hin zu einem Crash auslösen.
5. **Treppen **stellen selbst mit Unterstützung eine erhebliche Hürde dar.

**Pflegerische Maßnahmen**

- Aktionen jenseits der täglichen Routine und mit konkretem Crash-Risiko müssen sorgfältig geplant und gut vorbereitet werden, damit alles möglichst reibungslos verläuft.
- Für den Transport über kurze Strecken und innerhalb der Wohnung eignet sich ein ultraleichter Reise- oder Transitrollstuhl, sofern keine Treppen zu überwinden sind. Für kurze, liegende Transporte bieten sich Tragetücher und Transfertücher an. Diese sollten möglichst von vier Personen getragen werden, um Erschütterungen zu minimieren. Idealerweise wird der Einsatz zuvor mit einer anderen Person geübt, damit im Ernstfall jeder Handgriff sitzt.
- Für längere Transporte oder bei OI sollte ein Rollstuhl über eine Liegefunktion verfügen (Rückenlehne umklappbar und Beine hochstellbar).
- Ein Transport im Auto sollte möglichst liegend und mit offiziellem Krankentransport erfolgen. Spezielle Bedürfnisse wie Verdunkelung, Lärmschutz und schonende Fahrweise sollten im Vorfeld detailliert abgesprochen werden.
- Wenn verträglich und nach ärztlicher Verordnung, kann eine präventive Gabe von Sedativa (Beruhigungsmitteln) die Reizverarbeitung dämpfen und so das Risiko einer Zustandsverschlechterung reduzieren.
- Lagerungsinterventionen: Maßnahmen, auch zur Kontraktur- und Spitzfußprophylaxe, sollten nur erfolgen, sofern sie toleriert werden und keinen Crash auslösen durch Berührung, Bewegung oder Positionsveränderung. Wenn zur Dekubitusprophylaxe regelmäßiges Umlagern notwendig ist (im besten Fall durch Pflegefachpersonen eingeschätzt), müssen schonende Wendetechniken zur Druckentlastung und Vermeidung von Scherkräften erlernt und in die Routine eingebaut werden. Alle Pflegenden sollten dieselben Handgriffe und Abfolgen verwenden, um zusätzliche Belastungen möglichst gering zu halten. Bei generalisierter Gelenkhypermobilität können individuell angepasste Lagerungskissen oder -rollen zur Gelenkstabilisierung hilfreich sein (siehe dazu 3.4.5).
- Zur Sturzprophylaxe und Belastungsreduktion Hilfsmittel wie Rollstühle, Rollmobile, Gehilfen einsetzen und die Umgebung frei von Stolperquellen halten. Bei Bedarf können zusätzliche Sitzmöglichkeiten zur gezielten Entlastung eingesetzt werden.

**Hilfsmittel und praktische Tipps**

- Beim Kauf eines Rollstuhls auf Gewicht, Handhabbarkeit und möglichst vibrationsarme Fahreigenschaften achten. Bei schweren Rollstühlen (z. B. elektrisch) helfen Teleskopschienen bei kurzen Treppen oder beim Verladen. Für den Transfer zwischen Bett und Rollstuhl können Rutschbretter oder Gleitmatten nützlich sein.
- Treppensteighilfen ohne Umbau des Treppenhauses sind möglich, entweder als Geräte mit integriertem Sitz oder als Systeme, die einen Rollstuhl transportieren.
- Viele Hilfsmittel wie Rollstühle und Treppensteiger lassen sich auch mieten – ideal zum Ausprobieren oder für gelegentliche Nutzung.
- Not-Tragetücher oder Einmal-Transfertücher sind kostengünstig und sollten bei dauerhaft Bettlägerigen stets griffbereit gehalten werden, auch für eine schnelle Evakuierung, etwa bei akuter Brandgefahr.
- Wechseldruckmatratzen zur Dekubitusprophylaxe werden aufgrund der Geräuschbelastung und Druckveränderungen häufig nicht toleriert.

### 4.6 Sicherheit, Schutz und Infektionsprophylaxe

Bei schwerem ME/CFS umfasst Sicherheit mehr als klassische Sturz- oder Infektionsprophylaxe. Jede zusätzliche Belastung oder jeder Reiz, infektiologisch oder sensorisch, kann eine PEM-induzierte Zustandsverschlechterung auslösen. Sicherheit bedeutet daher in diesem Kontext vor allem: Schutz vor vermeidbarer externer Belastung bei gleichzeitiger Sicherstellung der Versorgung.

**Wesentliche Herausforderungen**

1. **Infektionen **stellen ein hohes Risiko für langfristige Zustandsverschlechterungen dar.
2. **Erkrankungen im Pflegeteam** können die Versorgungssicherheit erheblich gefährden.
3. Schwer Betroffene sind meist nicht in der Lage, **sich selbst ausreichend vor äußeren Reizen zu schützen.**
4. **Sensorische Reizempfindlichkeit** (Licht, Geräusche, Berührung, Gerüche, Temperatur, Chemikalien) kann extrem ausgeprägt sein und Pflegehandlungen stark erschweren.
5. **Reinigungs‑, Lüftungs- oder Instandhaltungsmaßnahmen** können selbst zur **Belastung **werden.

**Pflegerische Maßnahmen**

*Infektionsschutz und Expositionsprophylaxe:*

- Im Pflegezimmer tragen Pflegeteam und Besucher:innen konsequent eine gutsitzende FFP2- oder FFP3-Maske. Bei eigenen Krankheitssymptomen sollte der direkte Kontakt, wenn möglich, ganz vermieden und eine Vertretung organisiert werden.
- Auch außerhalb des Pflegezimmers auf Infektionsschutz achten (Masken in Innenräumen, gute Raumluft, empfohlene Impfungen).
- Konsequente Händedesinfektion vor jeder Pflegehandlung sowie vor Nahrungszubereitung.
- Zimmer soweit möglich regelmäßig lüften (unter Berücksichtigung von Licht- und Geräuschempfindlichkeit). Je nach individueller Toleranz kann ergänzend ein High Efficiency Particulate Air(HEPA)-Luftreiniger eingesetzt werden.
- Impfentscheidungen bei Betroffenen individuell unter Berücksichtigung von Vorerfahrungen und indirektem Schutz durch das Umfeld treffen.
- Sauberkeit im Pflegezimmer, Wechsel von Bettwäsche und Unterstützung bei der Körperpflege tragen zum Infektionsschutz bei, müssen jedoch stets an die Belastbarkeit angepasst werden.

*Schutz vor sensorischer Überlastung:*

- Sonnenbrillen oder Augenmasken sind oft trotz Abdunkelung notwendig und erhöhen die Toleranzgrenzen. Verschiedene Modelle ausprobieren und bei Masken ausreichend Wechselmöglichkeiten vorbehalten.
- Lärmschutz durch Kopfhörer oder Gehörschutzstöpsel müssen ebenfalls individuell ausprobiert werden. Noise-Cancelling-Kopfhörer können nützlich sein, werden aber nicht von allen Betroffenen toleriert (z. B. Druck auf Kopf und Ohren, eventuell verstärkter Tinnitus).
- Die Pflege muss häufig mit minimalen Lichtquellen auskommen und ggf. in totaler Dunkelheit erfolgen. Dies erfordert Übung und – hier ganz besonders – das Einhalten einer strikten Routine.
- Alltagsgeräusche so weit wie möglich reduzieren und notwendige geräuschvolle Arbeiten nicht während eines Crashs durchführen. Dennoch notwendige Maßnahmen vorher ankündigen und ein verlässliches Ende angeben (z. B. „5 min Bohren“). Intensive, aber kurze Lärmbelastung wird oft besser toleriert als lang anhaltende. In ärztlicher Absprache kann eine medikamentöse Unterstützung zur Reduktion der Reizbelastung erwogen werden.
- Keine Parfüms verwenden, Zimmer vor starken Gerüchen (Küche) möglichst abschirmen. Je nach Toleranz nur parfümfreie Waschmittel und Pflegeprodukte verwenden. Bei starker Unverträglichkeit gegenüber Chemikalien so weit wie möglich auf Wasch- und Putzmittel verzichten.
- Wenn eine Zimmerwahl möglich ist, ist eine ruhige Lage das wichtigste Kriterium (Geräusche und Stimmen innerhalb und außerhalb der Wohnung). Ebenfalls wichtig ist eine gute Verdunkelung und Belüftungsmöglichkeit zur Vermeidung von Schimmel. Eine Klimaanlage kann Temperatur und Luftfeuchtigkeit regulieren. Glatte Böden sind pflegeleichter als Teppiche.
- Die Reinigung sollte möglichst selten und so kurz wie möglich erfolgen, eventuell nur in kleinen Abschnitten von 1 bis 2 min. Für eine sehr gelegentliche Grundreinigung oder Umbauten ist, wenn verfügbar, ein Ausweichzimmer ideal, sofern ein Transfer (z. B. Rollstuhl, Tragetuch) möglich ist.

**Hilfsmittel und praktische Tipps**

- Ein individuell angepasster Gehörschutz kann eine große Hilfe sein. Die Anpassung (durch eine Fachkraft zu Hause) erfolgt durch Ausmessen des Ohres in wenigen Minuten. Es muss allerdings sowohl die kurze Prozedur als auch das notwendige Licht toleriert werden.
- Gezieltes Licht: Eine Stirnlampe mit fokussierbarem Lichtkegel ist praktisch für Aufgaben, bei denen gezielt nur ein kleiner Bereich beleuchtet werden soll, z. B. beim Schneiden der Zehennägel.
- Elektrische Rollläden oder fernbedienbare Systeme zur Regulierung von Licht, Temperatur und Luftfeuchtigkeit können Betroffenen ein Mindestmaß an Kontrolle und Selbstständigkeit ermöglichen. Eine frühzeitige Anpassung ist sinnvoll, solange der Zustand Veränderungen noch zulässt. Viele Maßnahmen lohnen sich bereits bei moderater Krankheitsschwere.

### 4.7 Kommunikation, soziale Interaktion, Menschen im Raum

Kommunikation ist ein unverzichtbares menschliches Grundbedürfnis. Bei schwerem ME/CFS sind die Möglichkeiten jedoch stark eingeschränkt und erfordern individuelle Anpassungen.

**Wesentliche Herausforderungen**

1. Die **Verarbeitung von Sprache** erfordert hohe kognitive Leistung und ist für schwer Betroffene oft noch belastender als einfache Geräusche.
2. Auch **Schrift** aktiviert das Sprachzentrum („man kann nicht nicht lesen“) und wird deshalb häufig als belastend empfunden.
3. Schon die reine **Anwesenheit von Personen** im Raum kann, selbst ohne Sprache, kognitiv anstrengend sein. Besonders gilt das für Besuche außerhalb der Routine, die starke Emotionen auslösen können (auch Freude kann belastend sein).
4. Betroffene im sehr schweren Spektrum sind oft **zu schwach zum Sprechen** oder Schreiben. Kommunikation erfordert zudem Konzentration, die oft nur eingeschränkt möglich ist.
5. **Crashgefahr durch Handynutzung**.
6. Gleichzeitig kann **vollständige soziale Isolation** psychisch sehr belastend sein.

#### Pflegerische Maßnahmen

Zunächst sollten die wichtigsten Kommunikationswege gesichert werden. Alle Maßnahmen gegen soziale Isolation müssen, soweit möglich, gemeinsam mit der betroffenen Person entwickelt und individuell abgestimmt werden.

- Das Handy ist für viele Betroffene die letzte, wertvolle Verbindung zur Außenwelt. Gerade deshalb ist ein Überschreiten der Toleranzschwelle eine große Gefahr. Eine zeitliche Begrenzung oder ein völliger Verzicht können notwendig sein. Die Entscheidung liegt, zumindest bei Volljährigen, allein bei den Betroffenen. In Absprache können technische Begrenzungen der Nutzungsdauer hilfreich sein.
- Wenn das Handy für die Kommunikation mit dem Pflegeteam verwendet wird, sollten Alternativen geschaffen werden, um einen zeitweiligen oder dauerhaften Verzicht möglich zu machen.
- Die Basiskommunikation sollte nonverbal erfolgen: z. B. Klingelknopf zum Herbeirufen einer Pflegeperson, Gesten, Fingerzeichen oder einfache Laute für „Ja“ und „Nein“. Wichtig ist ein eindeutiges Zeichen für „Stopp“, nach dem alle Tätigkeiten so schnell wie möglich abgebrochen werden und die Pflegeperson den Raum verlässt.
- Die Etablierung weiterer Zeichen ist sinnvoll und sollte bei Anzeichen von Sprachverlust rechtzeitig forciert werden, z. B. Zeichen für Durst (Trinken), Toilette, gut/schlecht, zu kalt/warm, zu laut/hell, Crash, Schmerzen, Danke. Auch für Emotionen (z. B. trauriger oder fröhlicher Smiley) und wichtige Personen (inkl. Pflegeteam und Ärzt:in) können individuelle Symbole genutzt werden. Bei der Kommunikation zu Körperstellen *(*Wo tut es weh?) eignet sich ein Avatar, etwa eine Puppe oder ein Stofftier mit Armen und Beinen. Bilder (z. B. kleine Täfelchen) sind oft besser geeignet als Schrift, die das Sprachzentrum belastet.
- Kommunikation seitens des Pflegeteams auf das Notwendige reduzieren: wenige klare Sätze oder einzelne Wörter, die vorher gut überlegt werden. Bei eingespielter Routine ist ein Gespräch über den Pflegeverlauf oft nicht notwendig. Wichtige Mitteilungen (z. B. Änderungen im Plan) können mit einzelnen Worten oder Zeichen übermittelt werden. Fragen als Ja/Nein-Fragen formulieren. Nicht notwendige Kommunikation (z. B. Grüße, Nachrichten aus der Welt) sollte nur zu geeigneten Zeiten und nach Aufforderung durch die betroffene Person erfolgen. Feste Zeiten für die Kommunikation helfen, dass sich Betroffene im Vorfeld darauf einstellen können, was die Belastung reduziert.
- Es sollten nie mehr Personen im Raum sein als notwendig. Bei sehr niedriger Toleranz müssen individuelle Lösungen gefunden werden: Soweit möglich, sollte die betroffene Person selbst essen oder sich mit Feuchttüchern waschen können, um zusätzliche Belastung zu vermeiden. Falls dies nicht möglich ist, kann die Pflege in Ausnahmefällen mit sedierenden Medikamenten erfolgen.
- Besuche sind anstrengend, manchmal aber wichtig, um die soziale Isolation zu durchbrechen. Die Entscheidung liegt ausschließlich bei den Betroffenen und muss akzeptiert werden. Besucher:innen sollten auf die Möglichkeit vorbereitet sein, dass sie kurzfristig doch nicht ins Zimmer können, selbst wenn sie dafür extra anreisen. Besuche können nur sehr kurz möglich sein und müssen ggf. ohne verbale Kommunikation auskommen. Sie sollten immer angekündigt werden – oft aber besser kurzfristig, um längere Anspannung zu vermeiden. Kurze, ritualisierte Abläufe (z. B. feste Besuchsdauer, klare Rollen, wenig Sprache) können helfen, die kognitive Belastung zu reduzieren und gleichzeitig durch Verlässlichkeit psychische Unterstützung bieten.

**Hilfsmittel und praktische Tipps**

- Eine Funkklingel mit mehreren Empfängern, die sich flexibel im Haus verteilen lassen, ist für den „Ruf“ meist eine gute Lösung.
- In extremen Fällen, wenn selbst das Drücken eines Klingelknopfs nicht mehr möglich ist, bietet ein Babyfon eine mögliche Alternative.

### 4.8 Umgang mit emotionaler und existenzieller Belastung

Schweres ME/CFS ist eine psychische Extremsituation. Die massive körperliche und kognitive Einschränkung, die dauerhafte Abhängigkeit von Hilfe, wiederholte Zustandsverschlechterungen sowie die Vernachlässigung der Erkrankung durch das Gesundheits- und Sozialsystem führen zu einer hohen emotionalen Belastung. Gefühle wie Verzweiflung, krankheitsbezogene Angst, Panik, Hoffnungslosigkeit oder Überforderung sind unter diesen Bedingungen häufige und erwartbare Reaktionen. Auch depressive Symptome können auftreten. Alle diese Symptome und Phänomene sind jedoch in der Regel Folge der anhaltenden Belastungen und nicht Ursache der Erkrankung. Wichtig ist eine klare Abgrenzung: Psychische Reaktionen dürfen nicht fälschlich als Erklärung für ME/CFS oder für Zustandsverschlechterung interpretiert werden.

Modelle wie das Vier-Phasen-Modell nach Fennell beschreiben Anpassungsprozesse im Verlauf chronischer Erkrankungen [40]. Bei sehr schwer Betroffenen ohne relevante Symptomverbesserung sind Integrations- oder Akzeptanzphasen (Fennell-Phasen 3 und 4) jedoch häufig nicht oder nur eingeschränkt erreichbar.

**Wesentliche Herausforderungen**

1. Psychische Belastungen sind, anders als viele körperliche Belastungen, oft kaum vermeidbar und zählen deshalb bei schwer Betroffenen zu den häufigsten Auslösern von Crashs.
2. Unkenntnis von ME/CFS und insbesondere von PEM bei Therapeut:innen oder im Umfeld kann zu Fehldiagnosen und schädlichen Interventionen führen (z. B. Aktivierung bei vermeintlicher oder auch tatsächlicher Depression).
3. Toxische Positivität („Du darfst die Hoffnung nicht aufgeben! Du musst kämpfen!“) verstärkt den Druck auf Betroffene und kann Schuldgefühle auslösen. Damit sind häufig Formen von „Victim Blaming“ verbunden, die implizite oder explizite Annahme, Erkrankte seien selbst für ihre Situation verantwortlich oder täten nicht genug. Auch im medizinischen Kontext kann dies als sogenanntes „Medical Gaslighting“ auftreten, wenn Beschwerden infrage gestellt oder bagatellisiert werden [41, 42]. Umgekehrt ist die Vorstellung einer vollständigen Akzeptanz der Situation als „höchste Verarbeitungsstufe“ bei schwerster Symptomatik kein realistisches Ziel.
4. Eingeschränkte Kommunikationsmöglichkeiten erschweren es Betroffenen, ihre emotionale Situation differenziert auszudrücken oder Hilfe einzufordern.
5. Phasen extremer oder lange anhaltender Belastung können existenzielle Krisen auslösen, auch Sterbe‑/Todeswünsche können geäußert werden.

**Pflegerische Maßnahmen**

- Emotionale Belastungen als relevante Crash-Auslöser berücksichtigen. Ziel ist der Schutz vor emotionaler Überlastung durch eine konsequente Begrenzung emotionaler Belastung im Rahmen der allgemeinen Belastungssteuerung (Pacing). Es geht nicht um die Bearbeitung oder Auflösung emotionaler Inhalte, sondern darum, ein von Tag zu Tag Aushaltenkönnen zu ermöglichen.
- Abschirmung vor belastenden Auslösern (Triggern), soweit möglich. Dazu zählen z. B. Ablehnungsschreiben von Behörden, belastende medizinische Informationen oder der Kontakt zu Personen ohne Krankheitsverständnis. Idealerweise erfolgt eine selbstbestimmte Abgabe solcher emotional fordernden Aufgaben an das Pflegeteam oder an Angehörige.
- Coping-Techniken, um mit der belastenden Grundsituation umgehen zu können. Bei schwer Betroffenen sind dies meist sehr niedrigschwellige Strategien, wie ritualisierte Tagesabläufe, kurze Momente positiver Zuwendung oder ein kurzes persönliches Mantra.
- Ruhiges, tiefes Atmen kann bei emotionalen Belastungen und Hyperventilation einen Beitrag zum Pacing leisten. Betroffene sollten sich ihrer Atmung bewusst sein und diese wenn möglich bei Bedarf eigenständig steuern können. Geeignete Interventionen sind oft minimal und flexibel, starre atemtherapeutische Techniken sind meist zu belastend und nicht umsetzbar.
- Medikamente (Antidepressiva oder in akuten Situationen Sedativa) helfen einigen Patient:innen. Ihr Einsatz muss ärztlich besprochen werden, idealerweise mit ME/CFS erfahrenen Ärzt:innen. Sie sind nicht als ursächliche Therapie zu verstehen, sondern sollten daran gemessen werden, ob sie zur Reduktion emotionaler Überlastung und damit zur Stabilisierung beitragen.
- Professionelle psychotherapeutische Unterstützung kann hilfreich sein, wenn
  - die betroffene Person dies wünscht,
  - die Therapeutin oder der Therapeut ME/CFS-Erfahrung hat und
  - die Kommunikations- und Belastungsgrenzen dies zulassen.
  Alle Interventionen müssen konsequent an der PEM-Problematik ausgerichtet sein und dürfen keine Zustandsverschlechterung auslösen. Aktivierende oder konfrontative Ansätze sind zu vermeiden.
- Supervision oder Fallbesprechungen für Pflegende können entlastend sein, insbesondere bei anhaltend belastenden Situationen oder bei Unsicherheit im Umgang mit existenziellen Themen.

#### 4.8.1 Umgang mit Todeswunsch

Bei sehr schwerem ME/CFS können extreme Belastungs- und Krisensituationen entstehen, in denen auch existenzielle Fragen und Wünsche thematisiert werden. In diesem Zusammenhang kann auch ein Todeswunsch auftreten. Erkenntnisse aus der Palliativversorgung zeigen, dass Todeswünsche in ihrer Intensität und Dauer variieren können und häufig ambivalente Anteile umfassen, wobei der Wunsch zu sterben und der Wunsch weiterzuleben gleichzeitig bestehen können [34]. Ein Todeswunsch kann sowohl situativ durch akute Belastung verstärkt werden als auch Ausdruck einer anhaltenden, existenziellen Belastungssituation sein. Letzteres ist bei schwerem ME/CFS der deutlich häufigere Fall. In diesen Situationen ist eine krisensensible, respektvolle Begleitung wichtig, ebenso – sofern verfügbar – die frühzeitige Einbindung palliativmedizinischer und unterstützender Fachstrukturen. Rechtliche und ethische Fragen werden im Rahmen dieses Leitfadens nicht vertieft, sondern sollten im Einzelfall mit den zuständigen Fachstellen geklärt werden.

- Aktiv nachfragen, wenn ein Verdacht besteht, ob Gedanken an den Tod, Suizidgedanken oder konkrete Pläne vorhanden sind. Es gibt keine Hinweise darauf, dass das Ansprechen solcher Gedanken diese erst hervorruft oder verstärkt [34].
- Nicht bewerten, nicht bagatellisieren, nicht überdramatisieren. Eine respektvolle, offene und empathische Haltung ist zentral. Es ist nicht notwendig, Antworten oder Lösungen zu liefern; auch das gemeinsame Aushalten ist eine Form wirksamer Begleitung [34].
- Bei Hinweisen auf akute Suizidalität (z. B. zunehmender Handlungsdruck, konkrete Pläne):
  - ernst nehmen,
  - Betroffene möglichst nicht ohne Unterstützung lassen, dabei die Belastungsgrenzen von Angehörigen berücksichtigen,
  - vorhandene Unterstützung (Angehörige, ärztliche Ansprechpersonen, Krisendienste) frühzeitig einbeziehen,
  - prüfen, ob belastende körperliche oder sensorische Faktoren reduziert werden können (vgl. [34]).
- Belastende körperliche Symptome als mögliche Auslöser berücksichtigen. Schmerzen, Atemnot, Unruhe, Angst oder schwere sensorische Überlastung können existenzielle Gedanken verstärken. Prüfen, ob hier eine bessere Symptomlinderung möglich ist.
- Bei der wiederholten Äußerung eines Todeswunsches ist es wichtig, relevante rechtliche Regelungen und Rahmenbedingungen zu kennen (z. B. zum Sterbeverfügungsgesetz, Verbot der Tötung auf Verlangen). Sie sollten jedoch nicht im Vordergrund stehen und nicht als unmittelbare Handlungsanweisung interpretiert werden. Stattdessen stehen ein respektvoller Umgang mit der Äußerung, die weitere Beobachtung sowie das Angebot von Gesprächen und unterstützenden Kontakten im Vordergrund.

***Krisentelefone und Notrufnummern in Österreich unter:*** www.gesundheit.gv.at/leben/suizidpraevention/anlaufstellen/notrufnummern.html ***.***

### 4.9 In Beziehung sein: Rollen in Familie und Partnerschaft

Schweres ME/CFS verändert grundlegende Rollen in Partnerschaft und Familie. Betroffene können nicht mehr wie gewohnt als Partner:in, Elternteil oder Familienmitglied präsent sein, während ihre primären Pflegepersonen neue Verantwortlichkeiten übernehmen. Dies kann Trauer, Schuldgefühle und Spannungen auslösen und sollte als Teil der pflegerischen Realität anerkannt werden. Formen des Umgangs müssen neu eingeübt werden. Liebe und Empathie gegenüber schwer an ME/CFS Erkrankten zeigen sich in der Verlässlichkeit, Sorgfalt und Ausdauer der (pflegerischen) Begleitung sowie in der konsequenten Unterstützung beim Krankheitsmanagement durch Pacing.

**Wesentliche Herausforderungen**

1. Die gewohnten **Regeln des liebe- und respektvollen Miteinanders **müssen bei schwerem ME/CFS zum Teil neu gelernt werden. Beziehungen werden auf die Probe gestellt und können zerbrechen, wenn sie sich nicht an die von der Krankheit vorgegebenen Rahmenbedingungen anpassen.
2. Die **Intensität der Einschränkungen** (physisch, kognitiv, orthostatisch, sensorisch, emotional) lässt im Gegensatz zu vielen anderen chronischen Erkrankungen kaum einen Raum für Beziehungspflege, der nicht von der Krankheit beeinträchtigt ist („Intrusiveness of ME/CFS“, [40]).
3. Das **Abhängigkeitsverhältnis in der Beziehung** Pflegeperson – Patient:in ist für beide Seiten herausfordernd.
4. **Konflikte** sind natürliche Elemente in jeder Beziehung, können bei schwerem ME/CFS aber nicht ausdiskutiert werden.
5. Berufliche und private Einschränkungen können für pflegende Angehörige einen **Verlust von gesellschaftlichem Status** bedeuten oder auf Unverständnis stoßen (Was hast Du davon?). Dies kann zu psychischer Belastung bis hin zu sekundärer Traumatisierung führen [40], oft mit Rückwirkungen auf die Betroffenen, die sich als Last empfinden.

**Pflegerische Maßnahmen**

- Vertrauen ist die Basis jeder Beziehung. Dafür ist es entscheidend, dass Betroffenen geglaubt wird. Dies gilt insbesondere bei der Auskunft über ihre Symptome. Patient:innen müssen sich darauf verlassen können, dass ihre oft eingeschränkte Kommunikation ernst genommen wird und dass Mitteilungen zuverlässig weitergegeben werden.
- Geborgenheit entsteht durch Zuverlässigkeit und Verständnis. Gute Pflegeroutinen sind dafür die Grundlage. Persönliche Rituale bieten darüber hinaus eine Möglichkeit, Vertrautheit herzustellen und gegenseitige Zuneigung auszudrücken. Rituale können verschiedene Rollen, zum Beispiel als Elternteil oder Partner, zum Ausdruck bringen oder Erinnerungen und Hoffnungen wachhalten. Sie sollten gemeinsam entwickelt werden und dürfen die Belastungsgrenzen nicht überschreiten. Bei Schwerstbetroffenen sind sie oft nonverbal und können auf einzelne Gesten oder Berührungen beschränkt sein, die in die Pflegehandlungen selbst eingeflochten sind.
- Vertrauenspersonen haben auch die Rolle von Beschützern und Pacing-Helfern. Besonders in emotional aufgeladenen Situationen oder bei kurzfristiger Aktivierung kann es vorkommen, dass Betroffene ihre Belastungsgrenzen überschreiten, ohne dies rechtzeitig wahrzunehmen. Erfahrene Pflegepersonen sollten sie dann darauf hinweisen und an die Einhaltung von Pausen erinnern. Man kann auch Regeln vereinbaren, wann an Pacing erinnert werden soll.
- Kontakt zu anderen ME/CFS-Betroffenen in ähnlicher Situation kann eine sehr wertvolle Ressource sein. In den schwersten Fällen ist dies aber höchstens sehr eingeschränkt möglich und erfordert die aktive Unterstützung des Betreuungsteams.
- Vertrauenspersonen sollten, soweit möglich, auch eigene Belastungen oder Kränkungen kommunizieren. Dabei eigene Gereiztheit möglichst außerhalb des Zimmers regulieren. Frust im Pflegeteam durch gegenseitige Unterstützung auffangen, einschließlich Ablösung bei akuter Überlastung, wenn möglich.
- Da die Pflege von ME/CFS-Betroffenen sehr langfristig sein kann, sollten sich pflegende Angehörige auch der eigenen Grenzen bewusst sein. Die Stabilisierung des Betreuungsteams ist für alle Seiten, auch für die Betroffenen, ebenso wichtig wie die medizinische Versorgung. Gute Kommunikation innerhalb des Teams und Vernetzung in Selbsthilfegruppen sind wesentliche Bausteine.

## 5. Hinweise für Pflegefachpersonen

Dieses Kapitel beschreibt die besonderen Anforderungen an die häusliche Versorgung von Menschen mit schwerem ME/CFS und stellt zentrale pflegerische Prinzipien, Strukturen und Handlungsempfehlungen dar. Es richtet sich in erster Linie an professionell Pflegende, bietet jedoch auch anderen Gesundheitsberufen eine praxisnahe Orientierung im Umgang mit dieser hoch vulnerablen Patient:innengruppe.

Die Pflege bei schwerem ME/CFS erfordert ein grundlegendes Umdenken gegenüber etablierten pflegerischen Konzepten. Im Zentrum stehen nicht Aktivierung und Ressourcenförderung, sondern die konsequente Vermeidung von Überlastung. Die PEM, die ausgeprägte körperliche Schwäche und Reizintoleranz definieren dabei den Rahmen allen pflegerischen Handelns. Maßnahmen, die in anderen Kontexten als förderlich gelten, können hier zu einer nachhaltigen Verschlechterung führen und dürfen nicht angewendet werden.

Stattdessen folgt die Versorgung den Prinzipien der Schadensvermeidung, Reizreduktion und minimalen Intervention. Dies umfasst insbesondere eine präzise Planung, eine stark reduzierte Interaktions- und Kommunikationsdichte sowie die konsequente Anpassung aller Maßnahmen an die individuell sehr engen und fluktuierenden Belastungsgrenzen der Betroffenen [4, 28]. Für formell Pflegende ist diese zurückhaltende Vorgehensweise häufig kontraintuitiv und emotional belastend [28]. Sie verlangt spezifisches Fachwissen, konsequente Selbstkontrolle und ein hohes Maß an Empathie. Ein an palliativen Pflegeprinzipien angelehnter Zugang kann in diesem Zusammenhang eine hilfreiche Orientierung bieten.

Nur unter diesen Voraussetzungen lässt sich eine pflegerische Umgebung schaffen, die Stabilisierung ermöglicht und in einzelnen Fällen auch funktionelle Verbesserungen zulassen kann. Trotz adäquater Versorgung bleibt der Krankheitsverlauf individuell unterschiedlich, und selbst bei optimalen Bedingungen kann keine Besserung garantiert werden [4, 12].

Die Pflege von schwer betroffenen ME/CFS-Patient:innen erfolgt – abgesehen von Notfällen – in der Regel in Form sehr kurzer, über den Tag verteilter Interventionen. Daraus ergeben sich besondere Anforderungen an Planung, Kommunikation und Koordination der Versorgung. Klare Strukturen, verlässliche Abläufe sowie personelle Kontinuität sind zentrale Voraussetzungen für eine sichere Versorgungssituation im häuslichen Setting. Ebenso wichtig ist eine vertrauensvolle Pflegebeziehung.

### 5.1 Aufbau einer vertrauensvollen Pflegebeziehung

Bei schwerem ME/CFS ist eine tragfähige und verlässliche Beziehung zwischen Pflegepersonen, Betroffenen und ihren Angehörigen zentral [4, 12, 43]. Da durch PEM eine hohe Vulnerabilität gegenüber Belastung und äußeren Reizen besteht, sind Betroffene in besonderem Maß auf die Sensibilität, Achtsamkeit und Anpassungsfähigkeit der Pflegenden angewiesen [12, 26, 43, 44]. Nur ein stabiler und vertrauensbasierter Beziehungsrahmen ermöglicht es, individuelle Belastungsgrenzen zu erkennen, pflegerische Maßnahmen sicher umzusetzen und belastungsinduzierte Verschlechterungen zu vermeiden [12, 26, 44, 45].

Die wichtigste Voraussetzung für eine solche Beziehung ist die Fähigkeit, Empathie, Aufrichtigkeit und Verlässlichkeit im pflegerischen Handeln zu vermitteln [12, 44]. Dazu gehören insbesondere die ernsthafte und wertfreie Anerkennung der berichteten Symptome sowie die konsequente Ausrichtung pflegerischer Maßnahmen an den individuellen Toleranzgrenzen der Betroffenen. Hierfür braucht es auch eine Bereitschaft, von gewohnten, standardisierten pflegerischen Abläufen abzuweichen [4, 12, 26, 44].

Ebenso erfordert eine tragfähige Pflegebeziehung einen pflegerischen Zugang, der sich an der Schwere und Komplexität der Erkrankung orientiert. Zentral ist dabei ein umfassendes Verständnis dafür, wie sich die Erkrankung auf alle Lebensdimensionen – körperlicher, psychischer, sozialer und auch existenzieller bzw. spiritueller Natur – auswirkt. Das beinhaltet auch, transparent mit den Grenzen des eigenen Wissens umzugehen und gleichzeitig Bereitschaft zu zeigen, sich fortlaufend zum aktuellen Stand der ME/CFS-Forschung und Leitlinien zu informieren [2, 12, 44].

Schweres ME/CFS kann einen vollständigen Verlust des früheren Lebens und der eigenen Identität bedeuten [12]. Hier benötigt es einen besonders achtsamen Umgang mit den Betroffenen und ihren Angehörigen, der Raum für Verlust und Trauer lässt und gleichzeitig Halt und Verlässlichkeit bietet. Pflegepersonen tragen maßgeblich dazu bei, dass Betroffene sich gehört, ernst genommen und unterstützt fühlen, was gerade vor dem Hintergrund wiederholter Erfahrungen von Bagatellisierung und Psychologisierung von hoher Bedeutung ist [26, 44, 46].

#### Angehörige und primäre Pflegepersonen als wichtige Ressource

Eine partnerschaftliche und vertrauensvolle Zusammenarbeit mit Angehörigen und primären Pflegepersonen hat in der häuslichen Versorgung von Menschen mit schwerem ME/CFS einen besonders hohen Stellenwert [4].

Primäre Pflegepersonen sind diejenigen, die – gemeinsam mit den Betroffenen – über längere Zeiträume hinweg individuelle Pflegeroutinen entwickeln. Ein differenziertes Verständnis der tagesabhängigen Belastbarkeit, der Frühzeichen von Überlastung sowie der individuell tolerierbaren Interventionen entsteht nicht im Rahmen einzelner Hausbesuche, sondern durch kontinuierliche Beobachtung, behutsames Erproben und wiederholte Anpassung im Alltag. Insbesondere bei sehr schwer Betroffenen können selbst basale Pflegemaßnahmen, wie etwa Körperpflege oder Lagerung, nur in kleinen Sequenzen mit längeren Erholungsphasen erfolgen. Eine solche individualisierte Versorgung lässt sich mit der strukturellen und meist sehr engen Taktung professioneller Pflegedienste kaum abdecken. Die Einbindung kontinuierlich anwesender Bezugspersonen – in der Regel Angehörige oder 24-Stunden-Betreuungspersonen – ist daher unverzichtbar [47].

Hinzu kommt, dass auch der direkte Austausch mit Betroffenen während eines Hausbesuchs häufig auf ein Minimum reduziert werden muss und in extremen Fällen kaum noch möglich ist. Die Kommunikation sollte in diesem Fall auf die wesentlichsten Fragen fokussieren. Auskünfte zu objektivierbaren Aspekten wie der Medikation oder über Beobachtungen zum Verlauf können Vertrauenspersonen (in der Regel pflegende Angehörige) übernehmen. Wichtig dabei ist, dass Betroffene selbst über die Kommunikationswege und ihre Vertretung entscheiden. Im besten Fall ist dies durch eine formelle Vollmacht dokumentiert.

Gleichzeitig benötigen auch Angehörige und primäre Pflegepersonen selbst Orientierung, fachliche Rückmeldung und emotionale Entlastung. Pflegende Angehörige von Menschen mit schwerem ME/CFS übernehmen häufig Aufgaben, die weit über klassische Unterstützungstätigkeiten hinausgehen. Sie koordinieren medizinische Termine, dokumentieren Medikation und Symptome, organisieren den Alltag, vertreten die Interessen der Betroffenen im Gesundheits‑, Sozial- oder Bildungssystem und leisten gleichzeitig kontinuierliche emotionale Unterstützung. Parallel erleben sie den Krankheitsverlauf mit seinen unvorhersehbaren und schweren Symptomen unmittelbar mit [4].

Hinzu kommen erhebliche gesundheitliche, soziale und finanzielle Belastungen, insbesondere im Kontext der häufig notwendigen Rund-um-die-Uhr-Betreuung. Viele Angehörige reduzieren ihre Erwerbstätigkeit oder geben diese vollständig auf, wodurch langfristige wirtschaftliche Unsicherheiten entstehen können. Gleichzeitig besteht durch die dauerhafte körperliche und emotionale Belastung ein erhöhtes Risiko für eigene gesundheitliche Beeinträchtigungen und psychosoziale Überlastung. Auch der Zugang zu Sozial- und Unterstützungsleistungen ist häufig erschwert, was die Situation zusätzlich verschärfen kann [4, 28, 48].

Vor diesem Hintergrund kommt der professionellen Pflege eine zentrale Rolle zu. Diese umfasst in erster Linie meist nicht die direkte pflegerische Versorgung selbst, sondern besteht aus koordinierenden, organisatorischen und beratenden Aufgaben. Anpassungen oder Veränderungen in den Pflegeroutinen sollten durch erfahrene Pflegepersonen begleitet, evaluiert und, wo erforderlich, neu angeleitet werden. Ebenso wichtig ist eine proaktive Wahrnehmung möglicher Überlastungssymptome bei pflegenden Angehörigen sowie – wenn möglich – die Vermittlung geeigneter Entlastungs‑, Beratungs- oder Unterstützungsangebote. Dies dient nicht nur dem Schutz der pflegenden Personen, sondern auch der Stabilität und Kontinuität der gesamten Versorgungssituation [4, 43].

### 5.2 Hausbesuch

Als Voraussetzungen für einen sicheren Hausbesuch bei Menschen mit ME/CFS gelten eine sorgfältige Planung, klare Kommunikation im Vorfeld und ein strukturiertes Vorgehen vor Ort.

Da die Belastbarkeit stark fluktuieren kann, müssen Zeitpunkt, Umfang und konkrete Durchführung der Maßnahmen am Tag des Hausbesuchs erneut überprüft und gegebenenfalls angepasst werden. Auch bei sorgfältiger Vorbereitung kann es erforderlich sein, Maßnahmen zu verkürzen, zu verschieben oder vollständig abzubrechen.

#### Planung und Vorbereitung

Vorab sollte geklärt werden, in welchem Umfang die betroffene Person selbst in organisatorische Entscheidungen einbezogen werden möchte und kann. Wenn möglich, sollte die Zustimmung und Mitbestimmung direkt eingeholt werden, etwa in Form einer kurzen schriftlichen Rückmeldung oder einer formellen Bevollmächtigung. In der Praxis erfolgt die Abstimmung häufig über Angehörige oder primäre Pflegepersonen, die mit der individuellen Situation vertraut sind (siehe 5.2).

Im Vorfeld sind insbesondere folgende Aspekte zu erheben:

- maximal tolerierbare Aufenthaltsdauer im Raum
- Lichttoleranz (Dunkelheit, schwaches Licht)
- Toleranz gegenüber Kommunikation
- Berührungstoleranz
- Toleranz gegenüber Gerüchen oder Personen im Raum

Diese Faktoren bestimmen Ablauf, Personalplanung und Materialvorbereitung. Alternative Kommunikationsformen – etwa schriftliche Mitteilungen oder die Weitergabe über Angehörige – sind in der Regel belastungsärmer als direkte Gespräche vor oder nach einer Pflegemaßnahme. Für den Hausbesuch sollte eine geeignete, an die Patient:innen abgestimmte Tageszeit gewählt werden.

#### Ablauf und Verhalten im Pflegezimmer

Am Besuchstag erfolgt vor Betreten des Zimmers eine letzte Abstimmung mit den primären Pflegepersonen oder – sofern möglich und gewünscht – mit der betroffenen Person selbst. Beleuchtung, Lagerung, Materialablage und Aufgabenverteilung sind eindeutig zu klären. Grundsätzlich gilt: So viel Vorbereitung wie möglich außerhalb des Zimmers durchführen, um die Aufenthaltsdauer im Raum auf ein Minimum zu reduzieren.

- Im Pflegezimmer sollte ruhig, strukturiert und zügig gearbeitet werden. Arbeitsschritte erfolgen in klarer Priorisierung und sinnvoller Reihenfolge, um Unterbrechungen zu umgehen. Wiederholungen oder Korrekturen sind nach Möglichkeit zu vermeiden.
- Ein eindeutiges Notsignal sollte vorab vereinbart werden, mit dem die betroffene Person eine beginnende Verschlechterung anzeigen kann (z. B. Handzeichen, Klingel, vereinbartes Wort). Wird dieses Signal gegeben, sind sämtliche Maßnahmen so rasch wie möglich zu beenden und der Raum ist zu verlassen.
- Invasive Maßnahmen (z. B. Blutabnahmen, Katheter- oder Sondenmanagement) sollten ausschließlich erfahrene und routinierte Pflegepersonen übernehmen. Personen in Ausbildung oder mit geringer praktischer Übung sollten bei ME/CFS Patient:innen nicht eingesetzt werden. Pflegende Angehörige sollte man nicht an der betroffenen Person selbst einlernen, sondern an einem Modell (z. B. für Sondenpflege).
- Erfahrungen aus vergangenen Interventionen sollten vorab eingeholt werden, um wiederkehrende Probleme möglichst zu vermeiden.
- Für invasive Pflegemaßnahmen kann, falls ärztlich indiziert, unterstützend eine Prämedikation eingesetzt werden.
- Umfangreichere Blutabnahmen können bei schwer Betroffenen eine erhebliche Belastung darstellen. Es empfiehlt sich folgende Vorkehrungen zu treffen [13]:
  - Blutabnahmen gut vorbereiten (inkl. Ersatznadeln und Röhrchen) und möglichst während der individuell „besten“ Tageszeit der Betroffenen durchführen.
  - Aufgrund des verminderten zirkulierenden Blutvolumens (dysautonom bedingt) auf zusätzliche Flüssigkeitssubstitution achten (1–2 Gläser Elektrolytgetränke, ggf. auch ungesüßtes Bio-Kokoswasser).
  - Bei stark ausgeprägter Schmerz- und Berührungsempfindlichkeit kann an der Punktionsstelle vorab ein Lokalanästhetikum aufgetragen werden.
  - Ein hypoallergenes Pflaster (oder Watte mit Micropore-Tape) an der Punktionsstelle verwenden.
- Ein strenger Infektionsschutz ist im Umgang mit ME/CFS-Betroffenen obligat. Die entsprechenden Maßnahmen dazu sind im Kapitel 4.6 nachzulesen.

### 5.3 Pflegeplan und Notfallmaßnahmen

Ein ausführlicher und gut strukturierter Pflegeplan bildet die Grundlage einer verlässlichen Zusammenarbeit zwischen den Patient:innen, dem primären Pflegeteam und formell Pflegenden. Ein Pflegeplan bei schwerem ME/CFS basiert auf den Erfahrungen und den daraus entwickelten Pflegeroutinen, die sich meist über längere Zeiträume hinweg zwischen den Betroffenen und ihren primären Pflegenden etabliert haben. Ziel ist, dass alle Pflegepersonen (informell und formell) die Pflegeroutine sicher umsetzen können, ohne an Betroffene Rückfragen stellen zu müssen. Selbst wenn Patient:innen noch sprechen können, sollte ihre Energie nicht für wiederholte Erklärungen aufgewendet werden müssen, sondern für zusätzliche, wirklich wichtige Mitteilungen zur Verfügung stehen.

Ein vollständiger Pflegeplan bei ME/CFS beinhaltet:

- Ein **medizinisches Assessment** mit den Diagnosen, Komorbiditäten, Hauptsymptomen sowie allen bekannten Faktoren, die Symptome auslösen, verschlechtern oder lindern können.
- **Persönliche Informationen** über die betroffene Person (z. B. als Einleitung), wie etwa Interessen, Hobbys und den früheren Beruf. Das hilft, ein realistisches Bild des Menschen zu vermitteln, der vor der Erkrankung aktiv war und am sozialen Leben teilgenommen hat. Auf diese Weise können Missverständnisse und falsche Vorstellungen leichter vermieden werden.
- Die **aktuelle Medikation**. Auch frühere Erfahrungen mit Medikamenten, einschließlich der Verträglichkeit.
- Im Hauptteil: Klare **Angaben zu ALLEN Pflegehandlungen** (Durchführungszeitpunkt, Reihenfolge und Ablauf), Medikamentengaben und Kommunikationsmöglichkeiten bzw. -mittel. Abläufe wie z. B. das Anreichen von Essen, Routinen für Toilettengänge oder konkrete Vorgehensweisen bei Schmerzen und anderen Symptomen sollen detailliert festgehalten werden.
- Ein Abschnitt für **Notizen **ermöglicht es, fehlende Punkte oder Änderungen festzuhalten. Der Pflegeplan sollte regelmäßig überprüft und bei Bedarf angepasst werden.

#### Notmaßnahmen und Interventionen

Ein eigener Abschnitt des Pflegeplans sollte klare Vorgehensweisen in Notfällen und nichtalltäglichen Belastungssituationen beinhalten. Dazu zählen zum Beispiel Hausbesuche durch Ärzt:innen, Therapeut:innen oder Gutachter:innen, aufwendige Pflegemaßnahmen mit hohem Crash-Risiko oder Krankentransporte.

- Für Notfälle muss vorab geklärt sein – sowohl im Betreuungsteam als auch mit der betroffenen Person – wer die **Hauptverantwortung **trägt und im Bedarfsfall Entscheidungen treffen darf. Diese Personen sollten verlässlich rund um die Uhr erreichbar sein.
- Es soll festgehalten sein, welche **Ärzt:innen mit Kenntnis der Situation** im Notfall kontaktiert (wann und wie) und ob im Bedarfsfall Hausbesuche gemacht werden können. Wenn sich Komplikationen abzeichnen, ist es oft sinnvoll, **frühzeitig Kontakt aufzunehmen**, um zu vermeiden, nachts oder am Wochenende auf einen Notdienst angewiesen zu sein, der die Situation nicht kennt.
- Für medizinisches Personal ohne Erfahrung mit ME/CFS kann ein **offizielles Informationsblatt** (z. B. [49]) hilfreich sein. Idealerweise wird dieses mit ärztlicher Unterstützung für den individuellen Fall ergänzt und angepasst, sodass wichtige Besonderheiten schnell ersichtlich sind.
- Ebenso muss klar sein, welche **Medikamente** in welcher Dosierung in Notfällen oder zur Vorbereitung belastender Maßnahmen gegeben werden dürfen. Häufig werden hierfür Benzodiazepine eingesetzt. Aufgrund möglicher Abhängigkeit, Nebenwirkungen und Gewöhnungseffekten sollte ihr Einsatz immer ärztlich abgesprochen werden. Auch für Transporte sollte im Vorfeld geklärt sein, ob eine Sedierung erforderlich ist und wie diese erfolgen kann.

#### Dokumentation des Krankheitsverlaufs

Da ME/CFS häufig mit starken Symptomschwankungen verbunden ist, ist eine systematische Dokumentation über den Verlauf sehr wichtig. Damit lässt sich auch beurteilen, ob beispielsweise ein neues Medikament Wirksamkeit zeigt oder ob bestimmte Pflegetätigkeiten (z. B. Haarewaschen) Auswirkungen auf die Betroffenen haben.

- Ideal ist eine **tägliche Erfassung** von Art und Schwere der Symptome sowie die Dokumentation von Crashs. Moderat Betroffene können dies oft noch selbst übernehmen in Form von Symptomtagebüchern. Bei Schwerbetroffenen geht diese Aufgabe an das Pflegeteam über.
- Individuell abgestimmt können zusätzlich Nahrungs- und Flüssigkeitsgaben, Änderungen in der Ernährung, Körpertemperatur und der Stuhlgang dokumentiert werden.
- **Reaktionen auf Medikamente und Nahrungsergänzungsmittel** sowie deren **Verlaufskontrolle** sorgfältig dokumentieren und im Pflegeplan aufnehmen. Hier empfiehlt es sich, (neue) Medikamentengaben zeitlich versetzt zu beginnen, um Reaktionen zuordenbar zu machen.

### 5.4 Elemente palliativer Pflegeprinzipien

Palliative Care beschreibt einen *„Ansatz zur Verbesserung der Lebensqualität von Patienten und ihren Familien, die mit Problemen konfrontiert sind, welche mit einer lebensbedrohlichen Erkrankung einhergehen. Dies geschieht durch Vorbeugen und Lindern von Leiden durch frühzeitige Erkennung, sorgfältige Einschätzung und Behandlung von Schmerzen sowie anderen Problemen körperlicher, psychosozialer und spiritueller Art“* [50].

Diese Definition bezieht Palliative Care auf Erkrankungen mit lebensbedrohlichen bzw. lebensverkürzenden Verläufen. Bei schweren Verläufen kann auch die Erkrankung ME/CFS zu einer lebensbedrohlichen Situation führen. Dennoch ist sie nicht primär durch einen terminalen Verlauf gekennzeichnet. Gleichzeitig stehen (Stand heute) keine kausalen oder kurativen Therapien zur Verfügung. Vor diesem Hintergrund und angesichts der komplexen Symptombelastung und der sehr herausfordernden Versorgungssituation bei Menschen mit schwerem ME/CFS stellt sich die Frage, inwieweit Haltungen und Ansätze aus der Palliative Care auch für diese Patient:innengruppe übertragbar und hilfreich sein können.

Die **grundlegende Haltung der Palliative Care** erscheint für die Versorgung von Menschen mit schwerem ME/CFS besonders gut geeignet. Diese Haltung ist von **Respekt, Wertschätzung und einer „radikalen Orientierung“ an den Bedürfnissen und Problemen** der Patient:innen geprägt [51]. Die Gründerin der Hospizbewegung, Cicely Saunders (1918–2005), hat mit dem Total-Pain-Konzept – einer ganzheitlichen Perspektive auf die Betroffenen – die Schmerztherapie nachhaltig verändert [52], ausgehend vom Gedanken, „Pain is what the patient says, it hurts“ [53]. Gerade angesichts der hohen Vulnerabilität und der großen Unterschiede in den Ausprägungen bei schwerem ME/CFS ist ein solcher uneingeschränkter Respekt vor dem individuellen Erleben der Betroffenen von zentraler Bedeutung (siehe dazu auch Abschnitt 5.1).

Ein weiterer wichtiger Aspekt von Palliative Care ist die **ganzheitliche Sichtweise** auf Patient:innen und das **Zusammenspiel verschiedener Berufsgruppen und Personen**. Im Umgang mit den Betroffenen – insbesondere angesichts existenziellen Leidens – erweist sich eine **mitfühlende und zugleich professionelle Haltung** als hilfreicher als eine distanzierte, primär hierarchisch geprägte Herangehensweise.

Darüber hinaus richtet Palliative Care den Blick ausdrücklich auch auf **Angehörige und das soziale Umfeld** [50]. Wie in Abschnitt 5.1 („Angehörige und primäre Pflegepersonen als wichtige Ressource“) dargestellt, tragen Angehörige bei ME/CFS häufig die Hauptlast der Versorgung und sind zugleich zentrale Wissensquellen im Versorgungsprozess. Ansätze, die ihren **Unterstützungsbedarf systematisch erfassen und stärken**, sind daher von besonderer Bedeutung [54].

Hand- und Lehrbücher für Palliative Care enthalten zahlreiche Impulse zum Umgang mit belastenden Symptomen und Situationen (siehe dazu: [55–57]). Zu beachten ist, dass palliative Versorgungskonzepte überwiegend im Kontext fortgeschrittener, häufig onkologischer Erkrankungen entwickelt wurden. Sie sind daher für ME/CFS nicht direkt übertragbar, können jedoch in angepasster Form wichtige Anknüpfungspunkte bieten. Für ME/CFS sind hier insbesondere Ansätze aus der **Symptomlinderung**, wie der Umgang mit Schmerz, Übelkeit oder krankheitsbezogener Angst, zu nennen. Auch die palliative Grundhaltung der „radikalen“ Orientierung an den Bedürfnissen der Patient:innen ist im Kontext der notwendigen **Reiz- und Belastungsreduktion** besonders anschlussfähig. Einige palliative Maßnahmen können daher nach sorgfältiger individueller Prüfung übernommen werden, viele andere erfordern aber zumindest eine Anpassung oder sind nicht geeignet. So können Interventionen, die auf Gesprächen aufbauen oder mit sensorischer Stimulation oder Berührung einhergehen (z. B. Aromatherapie) bei vielen Betroffenen PEM-induzierte Zustandsverschlechterungen auslösen und sind daher kontraindiziert. Entscheidend ist, dass auch Interventionen aus Palliative Care – wie alle Maßnahmen – konsequent an den individuellen Belastungsgrenzen ausgerichtet werden und sich am Pacing (Abschnitt 3.3) orientieren.

Ergänzend können Angebote aus der Hospizarbeit im Umgang mit spirituellen und existenziellen Fragen sowie spezifischen Belastungen in der Betreuung, sowohl für Betroffene (wenn möglich) als auch für Angehörige und primäre Pflegepersonen, eine unterstützende Rolle einnehmen (siehe z. B. Hospiz Österreich, [58]).

Auch Palliative-Care-Spezialist:innen aus unterschiedlichen Berufsgruppen verfügen über Kompetenzen, die für die Begleitung von Menschen mit schwerem ME/CFS grundsätzlich nutzbar sein können (vgl. [59]). Eine Konsultation kann sinnvoll sein, sofern Kenntnisse über ME/CFS und insbesondere über die Post-Exertionelle Malaise (PEM) vorhanden sind – dies sollte im Vorfeld abgeklärt werden. Eine Übersicht über Angebote in Österreich findet sich auf der Website von Hospiz Österreich [58].

Eine vertiefte Auseinandersetzung von Palliative-Care-Fachpersonen mit den Besonderheiten von ME/CFS könnte die Qualität der Versorgung zusätzlich verbessern. Der vorliegende Leitfaden kann hierfür eine erste Orientierung bieten.

**Tab. 1:** Ressourcen Myalgische Enzephalomyelitis/Chronisches Fatigue-Syndrom (ME/CFS) und Pflege

D‑A-CH Konsensus zu Diagnostik und Behandlung von ME/CFS [3]	Maßgeblicher deutschsprachiger Expert:innenkonsens zur Diagnostik und medizinischen Versorgung
NICE Guideline zu ME/CFS [2]	Britische Leitlinie zu Diagnose und Management; ergänzt durch praxisnahe Materialien für Social Care Professionals sowie für die umfassende medizinische Versorgung
Bateman Horne Center Clinical Care Guide [4]	Umfassender Leitfaden zu Diagnostik und medizinischer Versorgung, mit Kapiteln zu schwer und sehr schwer Betroffenen sowie pflegerischen Aspekten
Lehrbuch Palliative Care [55]	Praxisorientiertes, interdisziplinäres Lehrbuch zum Palliative-Care-Konzept mit Fokus auf patientenzentrierte Versorgung und Einbezug von Angehörigen und pflegenden Bezugspersonen

## Data Availability

Im Rahmen dieses Artikels wurden keine primären Daten generiert oder analysiert. Die zugrunde liegende Literatur wurde vollständig im Referenzverzeichnis aufgeführt. Es handelt sich um ein Konsensusdokument.

## Abkürzungen

hEDS: Hypermobiles Ehlers-Danlos-Syndrom
HSD: Hypermobilitäts-Spektrum-Störung
ICD-10: International Classification of Diseases, 10th Revision
MCAS: Mastzellaktivierungssyndrom
ME/CFS: Myalgische Enzephalomyelitis/Chronisches Fatigue-Syndrom
OI: Orthostatische Intoleranz
PEM: Post-Exertionelle Malaise
PoTS: Posturales orthostatisches Tachykardiesyndrom
SFN: Small-Fiber-Neuropathie
